# Choosing Variant Interpretation Tools for Clinical Applications: Context Matters

**DOI:** 10.3390/ijms241411872

**Published:** 2023-07-24

**Authors:** Josu Aguirre, Natàlia Padilla, Selen Özkan, Casandra Riera, Lídia Feliubadaló, Xavier de la Cruz

**Affiliations:** 1 Research Unit in Clinical and Translational Bioinformatics, Vall d’Hebron Institute of Research (VHIR), Universitat Autònoma de Barcelona, P/Vall d’Hebron, 119-129, 08035 Barcelona, Spain; aguirre.gomez.josu@gmail.com (J.A.); natalia.padilla@vhir.org (N.P.); selen.ozkan@vhir.org (S.Ö.); mcasandrariera@gmail.com (C.R.); 2 Hereditary Cancer Program, Program in Molecular Mechanisms and Experimental Therapy in Oncology (Oncobell), IDIBELL, Catalan Institute of Oncology, 08908 L’Hospitalet de Llobregat, Spain; lfeliubadalo@iconcologia.net; 3 Centro de Investigación Biomédica en Red de Cáncer (CIBERONC), 28929 Madrid, Spain; 4 Institució Catalana de Recerca i Estudis Avançats (ICREA), 08010 Barcelona, Spain

**Keywords:** clinical variant interpretation, molecular diagnostics, cost models, personalized medicine, in silico tools, pathogenicity prediction, classification with rejection, healthcare costs

## Abstract

Pathogenicity predictors are computational tools that classify genetic variants as benign or pathogenic; this is currently a major challenge in genomic medicine. With more than fifty such predictors available, selecting the most suitable tool for clinical applications like genetic screening, molecular diagnostics, and companion diagnostics has become increasingly challenging. To address this issue, we have developed a cost-based framework that naturally considers the various components of the problem. This framework encodes clinical scenarios using a minimal set of parameters and treats pathogenicity predictors as rejection classifiers, a common practice in clinical applications where low-confidence predictions are routinely rejected. We illustrate our approach in four examples where we compare different numbers of pathogenicity predictors for missense variants. Our results show that no single predictor is optimal for all clinical scenarios and that considering rejection yields a different perspective on classifiers.

## 1. Introduction

The clinical application of Next-Generation Sequencing (NGS) is currently limited by our inability to fully interpret its results [1]. Specifically, we cannot determine if the sequence variants detected through this methodology are benign or pathogenic with absolute accuracy. This problem, known as the Variant Interpretation Problem (VIP) [1], has important consequences in terms of patient lives and economic cost, and is considered one of the challenges determining the future of genomic medicine [2].

Pathogenicity predictors offer a promising solution for addressing the VIP in the case of missense variants, a common cause of inherited disease [3]. These predictors are bioinformatics tools that leverage machine learning algorithms to integrate various aspects of a variant’s impact, such as biophysical, biochemical, and evolutionary factors, to classify the variant as pathogenic or benign [4]. Fast and cost-effective, pathogenicity predictors have already been incorporated into biomedical research and clinical practice [5]. However, their large numbers (>50) and still-incomplete accuracies [4] pose a problem when the intended users have to find an adequate tool for their purposes.

Our research aims at finding a solution to the challenge of selecting a suitable pathogenicity predictor from multiple options for biomedical research and healthcare applications. To accomplish this objective, it is crucial to initially differentiate this challenge from the task of assessing the predictive performance of classifiers. Even though these two issues are connected, they are distinct and require different evaluations [6]. The performance of a classifier, as indicated by metrics like [7] the Area Under the Curve (AUC), Matthews Correlation Coefficient (MCC), sensitivity or specificity, demonstrates how well it addresses the technical or scientific issue it was designed for. Nevertheless, it is widely acknowledged [6] that these performance indicators do not necessarily reflect the applicability of a classifier. The applicability of a classifier quantifies the average consequences of employing the predictor in its intended operational context [6]. It takes into account the different outcomes and is especially vital in high-stakes situations, such as healthcare problems, where erroneous decisions can dramatically impact all stakeholders.

**Figure 1:**
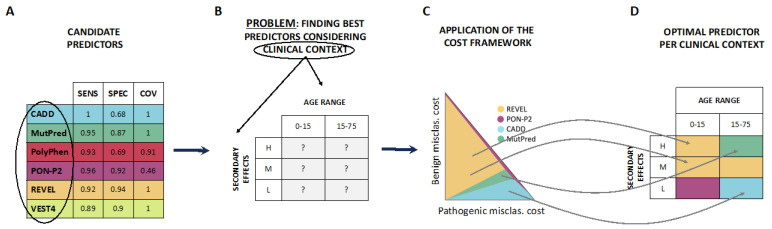
Context-dependent comparison of pathogenicity predictors. The figure illustrates the problem addressed in this work and outlines the solution we propose. (**A**). The starting point involves a set of pathogenicity predictors intended for a specific clinical application that requires variant classification as benign or pathogenic. Typically, these predictors are characterized by their specificity and sensitivity, representing their success rates in correctly identifying benign and pathogenic variants. Additionally, coverage indicates the fraction of variants for which the predictor can provide an outcome. (**B**). When utilizing pathogenicity predictors in clinical contexts, it is challenging to determine in advance which tool is preferable for specific circumstances. This is due to the fact that standard performance parameters (e.g., sensitivity, specificity) remain constant regardless of the clinical context of interest. In this example, the context is defined by the age range of the patient population (0–15 and 15–75) and the range of secondary effects of the existing treatment (H: High, M: Medium, L: Low). (**C**). This work introduces an innovative framework that describes the variability of application costs for different pathogenicity predictors across diverse clinical contexts. This framework can be applied to compare any number of tools. (**D**). The consistent results obtained from the four examples described in the article (see the Section 2) demonstrate that a single predictor valid for all clinical applications (or contexts) does not exist. On the contrary, the optimal predictor varies depending on the specific characteristics of the context.

This paper presents a framework to address the challenge of comparing multiple pathogenicity predictors in terms of applicability using cost models [8] (Figure 1). These models condense into a single value (the expected cost of using a predictor) the different external factors (or application context) relevant to a given application together with some specific properties of the considered tool [8]. The cost value enables interested users to evaluate all candidate predictors on a scale ranging from less to more optimal. In healthcare [9], the application context may include the impact of medical decisions on patients, their families, healthcare institutions, etc. The tool properties considered are the misclassification and rejection rates. An important characteristic of cost models is that the application context is summarized using only a few numerical parameters [9]. Note that in this text, we may interchangeably use the terms application context, clinical context, setting, or scenarios.

The framework presented in this study applies cost models to compare pathogenicity predictors while considering four key characteristics of the problem. Firstly, a general solution is necessary for comparing classifiers across different application scenarios. This is because healthcare scenarios vary significantly between and within countries in critical aspects of the application context, like providers and quality of care [10], and drug prices [11]. Secondly, multiple tool comparisons are required because many pathogenicity predictors (more than fifty) [4] are available to interested users. Thirdly, the cost associated with misclassification errors must vary depending on whether we are dealing with false positive or false negative classifications. This consideration is not routinely included in cost models [12]. Finally, it is essential to have a term taking into account the fact that rejecting computational evidence when the predictor is part of a medical decision pipeline [13] results in additional costs, including the need for more tests, patient travels, and other expenses.

Our approach starts with the cost model proposed for reject classifiers [14]. This model incorporates two opposing terms: one for error or misclassification rate and the other for rejection rate. In our study, we divide the misclassification rate into two terms: false positive and false negative classifications. The rejection rate accounts for the incomplete coverage of most prediction tools, especially when utilized within standard clinical guidelines [15]. We subsequently develop the framework for comparing multiple classifiers with a reject option across clinical scenarios. For comparison purposes, we derive the equivalent framework for the simpler case where the rejection term is disregarded. Finally, we employ our methodology to examine a set of seventeen pathogenicity predictors for missense variants (PolyPhen2-HDIV [16], PolyPhen2-HVAR [16], SIFT [17], CADD [18], MutationTaster2 [19], MutationAssessor [20], REVEL [21], LRT [22], PROVEAN [23], MetaLR [24], MetaSVM [24], VEST4 [25], MutPred [26], PON-P2 [27], SNAP2 [28], EVE [29], and PMut [30]). These predictors (described in the Section 4) classify missense variants as pathogenic, benign, or of unknown significance. They combine heterogeneous information sources to reach this goal [4], and while their overall accuracies may be easily above 80–90% [4], they present different sensitivity/specificity tradeoffs. This last aspect makes it difficult to choose the most appropriate method for a given application since sensitivity and specificity must be considered simultaneously in clinical applications [31]. Our results demonstrate how different methods may be favored depending on the clinical context, the impact of reduced coverage, and the sensitivity/specificity tradeoff. 

The work presented here is divided into two parts to enhance the manuscript’s readability. The first part establishes and explains the theoretical foundations of the cost framework and is presented in the Methods (Section 4.1 and Section 4.2) due to its methodological nature. We provide two versions of the cost framework: MISC (Section 4.1) and MISC+REJ (Section 4.2). MISC is a simplistic cost model that only takes into account misclassification errors when measuring the application cost of pathogenicity predictors. It is used to introduce some of the key concepts in cost models and as a reference for MISC+REJ. MISC+REJ, at the core of this work, is a more realistic model that takes into account the predictors’ rejection rate in addition to the misclassification errors. Readers who are less familiar with the formalism can skip the theoretical description of the cost models and proceed directly to the second part without sacrificing comprehension. In this second part, presented in the Section 2, we describe four applications of the cost framework. The first application explores the broader aspect of selecting the optimal predictor among a set of seventeen pathogenicity predictors. It examines how this choice is influenced by the clinical context and emphasizes the significance of considering rejection rates when comparing these predictors. In the second application, we narrow our focus to the *TP53* gene. Here, we demonstrate how the cost framework can uncover limitations associated with conventional performance measures like MCC when determining the most suitable predictor for this specific gene. This highlights the importance of adopting a comprehensive approach that goes beyond standard evaluation metrics. Additionally, two more examples are provided that delve deeper into the selection of pathogenicity predictors as a source of computational evidence for the ACMG/AMP guidelines. These examples further validate the key findings of this article, underscoring the substantial impact of clinical context on the optimal selection of pathogenicity predictors for deployment. 

## 2. Results

Here, we present four applications of our cost framework to select the best pathogenicity predictor among several options considering clinical context. In the first application (Section 2.1, Section 2.2 and Section 2.3), we describe how to use our methodology in a general version of the problem, where the number of pathogenicity predictors, seventeen, is high. Firstly, we start by computing some simple performance parameters (Section 2.1). Secondly, we show the use of the two versions of the cost framework: MISC (Section 2.2) and MISC+REJ (Section 2.3). In both cases, we describe how context may induce changes in the choice of predictors. As a reminder, readers can find a detailed description of these two cost frameworks in the Section 4. 

In the second application (Section 2.4), we show the use of the cost framework in the specific problem of choosing pathogenicity predictors for interpreting variants in the cancer-related gene *TP53*. Our results shed new light on the problem, showing that standard approaches are not optimal in all clinical scenarios. 

In the third application (Section 2.5), we analyze the outcome of the recently [21] proposed process for selecting pathogenicity predictors to classify variants using the ACMG/AMP guidelines. Our findings indicate that none of the methods universally apply to all clinical scenarios.

Finally, in the fourth application presented (Section 2.6), we employ the cost framework to contrast two suggested uses of computational tools in the two versions of the ACMG/AMP guidelines adapted to the *ATM* gene [15,32]. Consistent with our earlier findings, we discover that no single strategy is universally optimal across all clinical scenarios. 

### 2.1. Estimating the Sensitivity, Specificity, and Coverage/Reject Rate of the Pathogenicity Predictors

When comparing a set of predictors within the cost framework, the first step is to compute, for each predictor, three standard performance parameters: sensitivity, specificity, and coverage/reject rate (only for MISC+REJ). To this end, we utilized Equations (2a), (2b), and (6), respectively, along with the variants included in our dataset (see Section 4.3 in the Methods). 

The results obtained are listed in Supplementary Table S1. The coverage/reject rates deserve a comment. For the tools chosen for this work, the coverage varies between 0.43 and 1.0; correspondingly, the reject rates vary between 0.57 and 0. The predictors with coverage 1.0 (reject rate 0) are CADD, MutPred, REVEL, and VEST4. This is because for these predictors the authors do not provide any plug-in rule [14] (threshold(s) used to filter predictions based on their scores) to effectively transform these tools into classifiers with reject option. However, it must be mentioned that users or expert panels define plug-in rules adapted to their purposes when using these predictors in clinical applications. For example, in the variant interpretation guidelines for the *ATM* gene, ClinGen’s expert panel proposes [32] to use REVEL predictions as a source of computational evidence on a variant’s nature. However, not all the predictions are accepted; those with REVEL scores between 0.249 and 0.733 are discarded [32]. This plug-in rule is aimed at keeping highly reliable predictions only. A formal framework has been recently presented for the development and generalized use of these rules [33]. 

### 2.2. Application of the MISC Framework to the Comparison of Pathogenicity Predictors across Clinical Scenarios 

**Figure 2:**
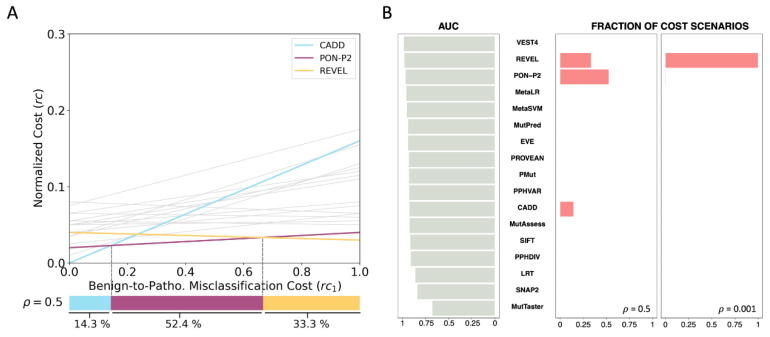
Application of the MISC framework to the comparison of pathogenicity predictors. In (**A**), the horizontal axis represents the clinical space (*rc*_1_ values corresponding to the costs of benign-to-pathogenic misclassification errors), while the vertical axis represents normalized costs, *rc*. Each line in (**A**) corresponds to one of the seventeen predictors compared using MISC. The colored lines denote the three pathogenicity predictors (CADD in light blue, PON-P2 in magenta, and REVEL in yellow) selected by MISC as optimal in specific clinical scenarios. The colored bars at the bottom of the figure depict the interval corresponding to each of these methods; the percentages below are a relative measure of size. The gray lines correspond to those methods that are never cost-optimal. (**B**). The figure demonstrates the impact of context on the evaluation of pathogenicity predictors for clinical use by comparing AUC (grey bars) and cost models (pink bars; outcomes for *ρ* values of 0.5 and 0.001). The seventeen pathogenicity predictors are ranked according to their respective AUC values, which are independent of the clinical context. Thus, the resulting ranking, VEST4 first, REVEL second, etc., is constant, regardless of the clinical context. This picture contrasts with the context-aware view offered by the pink bars, which indicate which method outperforms the others in terms of cost (the bar size indicates in how many scenarios). Remarkably, VEST4 is not optimal in any cost scenario, while CADD is represented in a fraction of cases when the benign-to-pathogenic misclassification costs are low.

To compare pathogenicity predictors using MISC, the first thing we must notice is that each pathogenicity predictor is characterized by a line relating normalized cost (*rc*) and clinical scenario (represented by its *rc*_1_ value) (Equation (4)). Figure 2A shows the lines for the seventeen predictors analyzed for *ρ* = 0.5. At each clinical scenario (*rc*_1_ value), the preferred predictor, in terms of cost, will correspond to the line with the lowest *rc*. 

To generalize this comparison to all possible clinical scenarios (i.e., *rc*_1_ values), we follow the procedure outlined in the Methods Section 4.1.2 ‘Predictor comparison across clinical scenarios’. Our analysis reveals that the clinical space can be divided into three sub-intervals, depicted in the form of three adjacent colored bars in Figure 2A (bottom). The sizes of these bars indicate the fraction of clinical scenarios where the associated pathogenicity predictors (CADD, PON-P2, and REVEL) are preferred. Above each interval, we observe that the line of the respective predictor occupies the lowest position relative to the other lines. In essence, we see that CADD, PON-P2, and REVEL provide a solution to the challenge of selecting the preferred tools across the clinical space.

An important aspect of this finding is that no single pathogenicity predictor is cost-optimal across all clinical contexts; rather, the optimal tool depends on the specific clinical scenario. This is in contrast to the results we would obtain if we ranked the tools based on their predictive performance metrics (e.g., AUC, MCC, etc.). To demonstrate this point, we sorted the seventeen predictors based on their AUC (Figure 2B) and found that VEST4 was the top-ranking predictor and, therefore, the tool of choice based on this criterion. However, from a cost perspective, there is no scenario where VEST4 is preferred over the other predictors. While using MCC instead of AUC yields a better coincidence, the underlying problem persists (Supplementary Figure S1A). 

**Figure 3:**
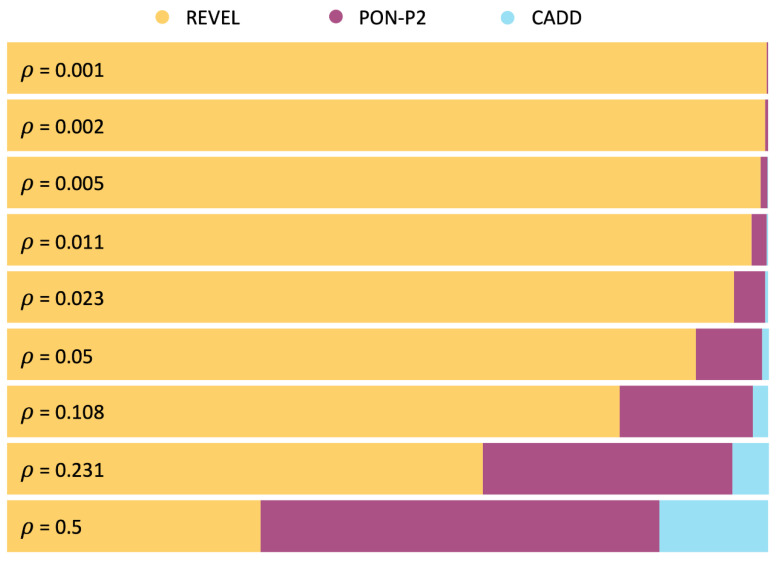
Dependency on *ρ* of the distribution of pathogenicity predictors in the clinical space, under the MISC framework. The frequency of pathogenic variants in the sequenced region, *ρ*, is a component of the cost model (see Equation (1)) that reflects the impact of the population context. Therefore, its values may affect the selection of pathogenicity predictors. We systematically explore the influence of *ρ* on the comparison of seventeen pathogenicity predictors. Each horizontal bar in the figure represents a partition of the clinical space, similar to the bottom bar in Figure 2A. The colored segments within each bar correspond to different methods, indicating the proportion of the clinical space where they are predominant. For instance, the top bar in the figure corresponds to a *ρ* value of 0.001, and we observe that the REVEL pathogenicity predictor (yellow) dominates almost the entire clinical space. As *ρ* values increase, two other pathogenicity predictors emerge, PON-P2 (magenta) and CADD (light blue).

In closing this section, we would like to address the impact of *ρ*, the frequency of pathogenic variants, on the tool selection problem. This parameter reflects the population context of the sequencing experiment. As an integral part of the cost formula, this parameter influences the selection of the preferred predictor. For instance, if we modify *ρ* from 0.5 to 0.001, we find that REVEL dominates the other methods, being preferred in 99.8% of the cost scenarios (Figure 2B). In presenting the findings of the MISC model, we have utilized *ρ* = 0.5, which assumes that the patient population is a biased sample of the general population, and thus the fraction of pathogenic variants is expected to be closer to that of benign variants. However, users may explore other *ρ* values, depending on their application of interest. In Figure 3, we examine the impact of different *ρ* values on the partition of the clinical space between methods. As we move from low to high *ρ* values, we observe a transition from REVEL to PON-P2, reflecting the higher sensitivity of PON-P2 (0.96) compared to REVEL (0.92). In summary, we find that *ρ* has a significant impact on the problem of finding the best pathogenicity predictor among a set of options.

### 2.3. Application of the MISC+REJ Framework to the Comparison of Pathogenicity Predictors across Clinical Scenarios

In this section, we present the application of MISC+REJ to the comparison of seventeen predictors taking into account clinical context. This application is more realistic than that of MISC because most of these predictors function as classifiers with a reject option or are employed as such through user-derived thresholds. 

**Figure 4:**
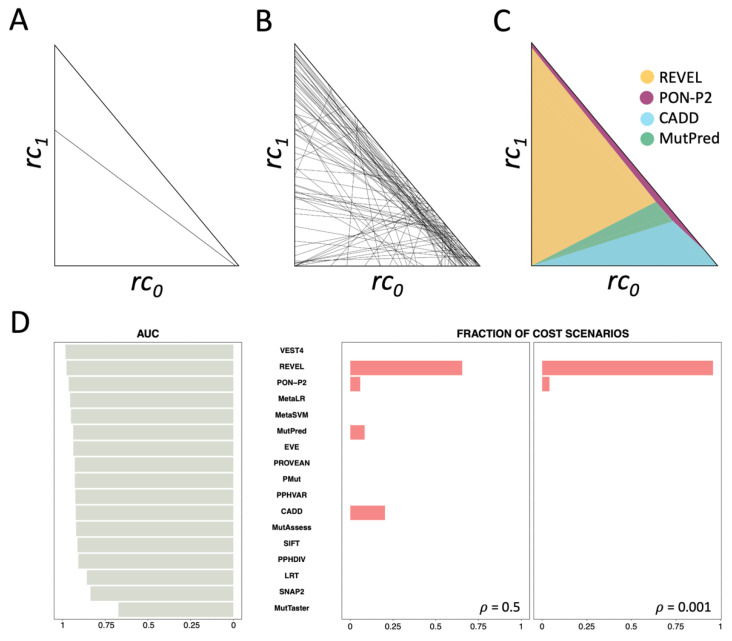
Application of the framework MISC+REJ to pathogenicity predictors. In (**A**,**B**), we show the results of comparing two and seventeen pathogenicity predictors, respectively, in MISC+REJ. These comparisons generate a series of polygons that usually need to be merged to obtain the regions of the clinical space (triangle ***T***, see (**A**–**C**)) where a predictor is dominant over others. In (**C**), we show the results of this unification using our adapted Breadth First Search algorithm. Note. The lines in (**A**–**C**) have a different meaning from those in Figure 2A. (**A**). The comparison of two predictors in MISC+REJ results in a dividing line that typically separates ***T*** into two regions, each with a cost-preferred predictor. The figure illustrates these regions, with one above and the other below the boundary line that traverses ***T***. No further processing is required in this case. (**B**). Comparison of seventeen predictors. Each line corresponds to a pair comparison. The outcome of all feasible comparisons between predictors generates a complex set of polygons. Manually exploring these polygons to determine the cost-optimal predictor is not feasible. (**C**). Our adapted Breadth First Search technique explores the intricate polygon pattern in (**B**) combining the polygons into regions where the same predictor is the preferred choice. These regions are colored based on the prevailing pathogenicity predictor: REVEL (yellow), CADD (light blue), MutPred (green), and PON-P2 (magenta). (**D**). This figure, equivalent to Figure 2B for MISC, demonstrates the impact of context on the evaluation of pathogenicity predictors for clinical use by comparing AUC (grey bars) and cost models (pink bars; outcomes for *ρ* values of 0.5 and 0.001). The seventeen pathogenicity predictors are ranked according to their respective AUC values, which are independent of the clinical context. Thus, the resulting ranking, VEST4 first, REVEL second, etc., is constant, regardless of the clinical context. This picture contrasts with the context-aware view offered by the pink bars, which indicate which method outperforms the others in terms of cost (the bar size indicates in how many scenarios). Interestingly, VEST4 is not optimal in any cost scenario, while CADD is represented in a fraction of clinical scenarios.

The goal here is to show the regions of the clinical space (***T***, a triangle in the *rc*_0_*-rc*_1_ plane, Figure 4A) where the different predictors are cost-optimal. In cost terms, the preferred predictor for a given pair (*rc*_0_, *rc*_1_) corresponds to the method with the lowest *rc* value (as specified in Equation (8)). To extend this comparison to all possible clinical scenarios ((*rc*_0_, *rc*_1_) pairs), we employ the procedure outlined in the Methods Section 4.2.2 ‘Predictor comparison across clinical scenarios’. This process divides the clinical space into a pattern of polygons (as illustrated in Figure 4B) that, after polygon unification, results into four regions (depicted in Figure 4C), corresponding to the predictors REVEL, CADD, MutPred, and PON-P2. The surface area of these regions is proportional to the fraction of clinical scenarios where the corresponding methods prevail. These outcomes are obtained for *ρ* = 0.5.

The result is noteworthy as it aligns with MISC’s results (previous section), according to which there is no single cost-optimal predictor suitable for the entire clinical space. Rather, the most effective predictor varies depending on the unique clinical context. Naturally, MISC+REJ shows some clear differences relative to MISC. In some scenarios, a new cost-optimal method, MutPred, emerges while there are significant changes in the rankings of the remaining three. Notably, REVEL now outperforms PON-P2 in more scenarios. This reversal is largely influenced by the rejection rate, as both predictors have similar sensitivities and specificities (Supplementary Table S1). However, we utilized REVEL with a 0% rejection rate, whereas PON-P2 had a rejection rate of 54%.

These results emphasize the idea that if we solely rely on performance metrics to evaluate predictors, we may overlook essential information. This becomes particularly clear when we examine the classification of predictors based on AUC as before. We can see (Figure 4D) that VEST4, which holds the highest ranking in terms of AUC, is not the preferred choice in any clinical scenario. Excluding VEST4, the match improves as the second predictor in the AUC ranking, REVEL, is associated with more scenarios (Figure 4C). However, CADD, which dominates in 20% of the scenarios, is only ranked eleventh in AUC classification (Figure 4D). The same analysis with MCC (Supplementary Figure S1B) demonstrates that this metric also fails to capture the REVEL-PON-P2 reversal observed with cost models. Moreover, the outcome for CADD is similar to that observed with AUC.

**Figure 5:**
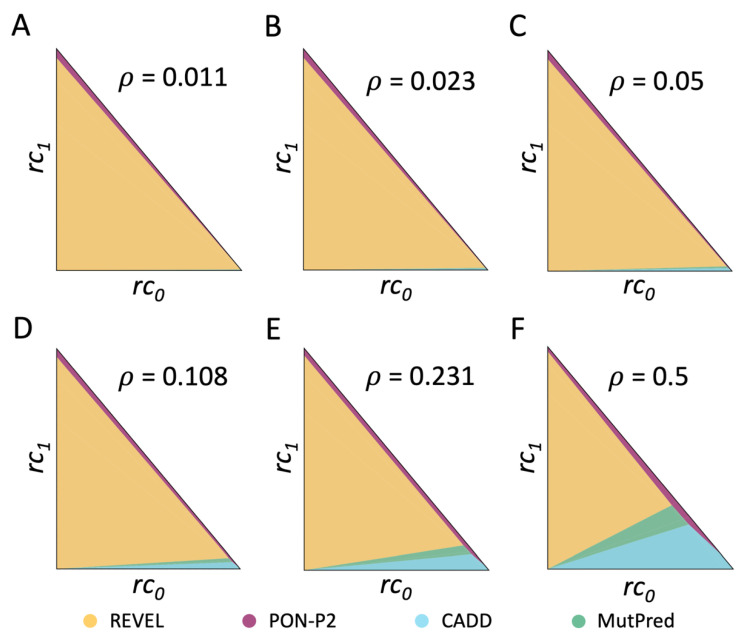
Dependency on *ρ* of the distribution of pathogenicity predictors in the clinical space, under the MISC+REJ framework**.** The frequency of pathogenic variants in the sequenced region, *ρ*, is a component of the cost model (see Equation (8)) that reflects the impact of the population context. Therefore, its values may affect the selection of pathogenicity predictors (the triangles in (**A**–**F**) figures are obtained with increasing values of *ρ*). Here, we systematically explore the influence of *ρ* on the comparison of seventeen pathogenicity predictors. The analysis in this figure is equivalent to that of Figure 3. However, the clinical space is represented here by the triangle ***T***. Each triangle represents a partition of ***T*** corresponding to a given *ρ* value. The colored regions within the triangles group to the cost scenarios where the same predictor is cost-optimal: REVEL (yellow), PON-P2 (magenta), CADD (light blue), and MutPred (green). Overall, REVEL tends to prevail over the remaining methods, although as *ρ* values increase, other pathogenicity predictors are also represented in some clinical scenarios.

Finally, we investigate the impact of *ρ*, the frequency of pathogenic variants, on the tool selection problem in the MISC+REJ model. As demonstrated in Figure 4D, using *ρ* = 0.001 instead of *ρ* = 0.5 does not significantly modify the results for REVEL, which prevails over most clinical scenarios. CADD and MutPred are no longer selected. Examining the outcomes for different *ρ* values (Figure 5), we observe a clear predominance of REVEL. This predominance has multiple origins. One is the zero rejection rate of REVEL in contrast to PON-P2. The second is its superior specificity (0.94) relative to CADD (0.68) and MutationTaster2 (0.87). This effect is evident as we observe the surface area occupied by CADD and MutationTaster2 expand as the fraction of benign variants decreases, that is, as *ρ* rises from 0.01 to 0.5. We can conclude that, as for MISC, the value of *ρ*, which captures the population context of the sequencing experiment, also has an impact on the problem of finding the best pathogenicity predictor, among a set of options.

To conclude this section, it is worth mentioning that when evaluating the results from the perspective of users interested in finding candidate predictors for specific clinical scenarios, a notable trend of REVEL prevailing in many of them is observed. However, it is essential to note that no predefined rejection region was applied to REVEL in this specific analysis, i.e., no predictions were rejected based on their scores.

### 2.4. Cost Analysis for TP53 Gene Computational Evidence Criteria

**Figure 6:**
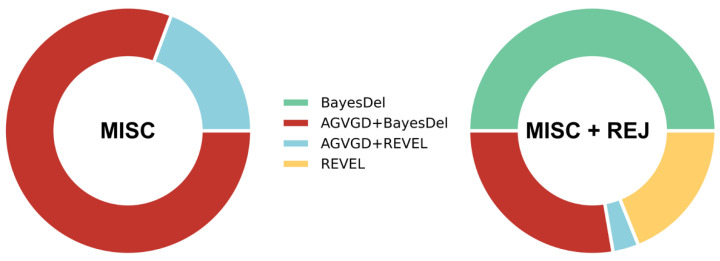
Application of the MISC and MISC+REJ frameworks to the eleven predictors in the Fortuno et al. [36] study. The colored regions in each ring represent the fraction of the clinical scenarios where each method is cost-optimal. Firstly, neither of the frameworks showed that the predictor recommended by Fortuno et al., AGVGD+BayesDel (red), is always the most cost-effective option. In the MISC model, the combination of AGVGD+REVEL (light blue) emerged as an alternative in some scenarios; in the MISC+REJ model, three predictors together, namely BayesDel (green), REVEL (yellow), and AGVGD+REVEL (light blue), covered more scenarios than ALIGN+BayesDel (red). Secondly, the comparison of MISC (left) and MISC+REJ (right) results shows that taking into account the rejection rate of predictors can significantly alter the selection of cost-optimal predictors for *TP53*.

Here, we illustrate the applicability of the MISC+REJ framework focusing on the case of the *TP53*-adapted guidelines for interpreting sequence variants in a clinical setting [34]. Typically, expert panels produce such adapted guidelines [35], which provide healthcare professionals with recommendations on which pathogenicity predictors to use and how to combine their results. In this case, the expert panel recommended [34] the combination of predictors Align-GVGD+BayesDel for the classification of *TP53* missense variants, based on a study [36] that used MCC to compare eleven predictors and predictor combinations. We obtained the *s_e_*, *s_p_*, and *ρ* of the predictors tested from the original paper [36] and compared these tools using the MISC and MISC+REJ frameworks (Figure 6 and Supplementary Figure S1C,D). Our findings are consistent with the results in the previous sections, indicating that no single predictor is optimal across the entire clinical space. Both the MISC and MISC+REJ analyses indicate that, in some scenarios, AGVGD+BayesDel has more cost-optimal alternatives. In MISC (Figure 6, left), the combination of AGVGD+REVEL emerges as an alternative in some cases. In the more realistic MISC+REJ (Figure 6, right), three predictors, BayesDel, REVEL, and AGVGD+REVEL, are now covering more scenarios than ALIGN+BayesDel. 

The differences between the results for MISC and MISC+REJ also highlight the importance of considering the rejection rate when selecting a pathogenicity predictor, as it can impact our view on which method is optimal. 

### 2.5. Cost Analysis of the Pathogenicity Predictors Studied by Pejaver et al. [33]

In a recent study, Pejaver and colleagues [33] conducted a comprehensive analysis of thirteen pathogenicity predictors (see Supplementary Table S2) as sources of computational evidence to support the interpretation of missense variants using the ACMG/AMP [5] guidelines. The authors of this study present thresholds for each predictor that determine the level of evidence associated with its prediction scores. For example, REVEL scores between 0.003 and 0.016 correspond to a level of ‘strong’ evidence of benignity. The authors find that for some tools they can define more evidence levels than for others, making their use more desirable, particularly when other sources of clinical evidence are scarce. Here, we use our cost framework to compare the thirteen methods and see whether some of them predominate over the clinical space because this could help in the tool election process. 

To apply our MISC+REJ model we have computed the sensitivities, specificities, and reject rates for each of the thirteen predictors (Supplementary Table S2) using a variant dataset provided by Pejaver et al. [33].

**Figure 7:**
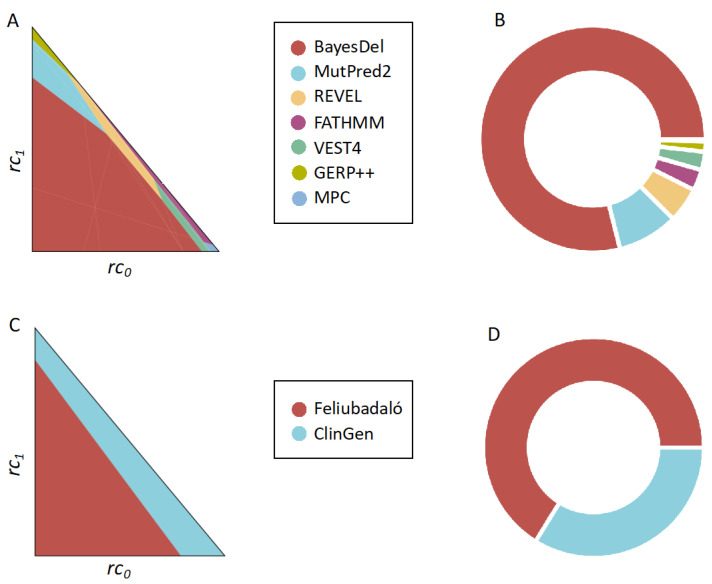
Application of the MISC+REJ framework to compare predictors within the context of the ACMG/AMP guidelines. In (**A**,**B**), we present the comparison of the thirteen predictors studied by Pejaver et al. [33]. Interestingly, only seven of these predictors are considered cost-optimal in at least one clinical scenario. (**A**) depicts these scenarios using a triangle, where each point represents an (*rc*_0_, *rc*_1_) pair (a specific clinical scenario, see text). On the other hand, (**B**) represents the raw count of scenarios in which each predictor prevails. Notably BayesDel emerges as the predominant predictor in the majority of cases. In (**C**,**D**), we conduct a similar analysis to evaluate the source of computational evidence in the two *ATM*-adapted ACMG/AMP guidelines [15,32]. We use the same representations as in (**A**,**B**). We observe that the two approaches compared exhibit prevalence in different regions of the clinical space. A common theme emerges from this figure, consistent with the previous examples: there is no single approach for computational pathogenicity prediction/annotation that prevails across the entire clinical space.

The results obtained are presented in Figure 7A,B. We see that seven out of the thirteen methods are cost-optimal in some clinical scenarios, although their distribution across the clinical space is diverse. For example, BayesDel is the dominant method in 79% of the clinical scenarios, while REVEL and MutPred2, which have more evidence levels in Pejaver et al. [33], dominate in 5% and 8.7% of the clinical scenarios, respectively. This difference is partly attributable to the difference in rejection rates between BayesDel (20%) and Revel (27%) and Mutpred2 (23%), because their sensitivities and specificities are closer to one another (Supplementary Table S2). 

These findings on the applicability of pathogenicity predictors align with the trend observed in the *TP53* analysis (Figure 6), where BayesDel stands out in multiple scenarios when assessed with the realistic MISC+REJ cost model. It is worth noting that the stringent rejection region defined by Pejaver et al. [33] for REVEL significantly reduces its applicability relative to other tools (e.g., compare Figure 2 and Figure 7A,B), especially in clinical scenarios with high costs associated with rejecting predictions.

### 2.6. Cost-Based Comparison of the Computational Evidence Used in the Two ATM-Adapted ACMG/AMP Guidelines

Recently, two modified versions of the ACMG/AMP guidelines, specifically tailored for the *ATM* gene, have become available [15,32]. Each version offers a unique approach to incorporating in silico tools into the clinical interpretation of missense variants in this gene.

The first approach, developed by Feliubadaló et al. [15], combines the outcomes of pairs of pathogenicity predictors in a sequence-dependent manner. This allows for the classification of variants as pathogenic/benign or the rejection of computational evidence when discrepancies arise. The second approach, developed by a ClinGen expert panel [32], relies on defining rejection regions based on the values of the REVEL [21] score. In this case, the REVEL predictions are rejected as evidence for interpretation when the REVEL score falls between 0.249 and 0.733. The corresponding sensitivities, specificities, and rejection rates are provided in Supplementary Table S3.

To compare these two approaches and explore their respective advantages in different clinical contexts, we applied our MISC+REJ formalism. Our results (Figure 7C,D), consistent with the findings from earlier sections, indicate that the clinical context significantly influences the preference for either version of the *ATM*-adapted guidelines. Specifically, we observe that ClinGen’s version predominates nearby the diagonal region of the cost space, where the relative rejection costs (*rc*_2_, see above) are smaller. This preference arises because the rejection rate is approximately 17%, higher than the rejection rate of Feliubadaló’s version, which is approximately 5%. However, as rejection costs increase (moving from the diagonal towards the lower-left vertex of the triangle), Feliubadaló’s version, with its lower rejection rate, becomes more cost-optimal compared to ClinGen’s version of the *ATM*-adapted guidelines. 

If we consider these results from the point of view of identifying the best computational source of evidence for the ACMG/AMP guidelines [5], we see that the combination of two predictors proves to be a competitive strategy for *ATM*. This approach outperforms the use of a single predictor, such as REVEL with a stringent rejection region, in regions of the clinical space where rejection costs are high. However, when combined, these two options provide comprehensive coverage across the entire clinical space.

## 3. Discussion

This manuscript addresses a relevant problem in the clinical classification of genetic variants: the selection of the most suitable pathogenicity predictor from a wide variety of candidates, taking into account the diversity of deployment contexts. Our cost-based framework (MISC+REJ), constituted by a formal core and computational solution (whose Python version is freely available), offers an initial response to this problem by addressing its two primary aspects. Firstly, it models the existing diversity of clinical scenarios that, when overlooked, can lead to unsuitable predictor recommendations for medical communities with resource constraints differing from those of the average community. Secondly, it considers pathogenicity predictors as predictors with a reject option, consistent with their typical use in clinical settings. 

Our approach is based on the cost models commonly employed in classifier evaluation [37] because their parameters can capture crucial aspects of the medical decision-making process (e.g., cost of missing patients, cost of treating healthy individuals, etc.) [9]. In this context, the solution to the classifier comparison problem involves determining the cost-optimal distribution of classifiers across the clinical space, where each point represents a specific clinical scenario. In the case of MISC+REJ, solving this problem requires partitioning the two-dimensional clinical space, which is a challenging task that must be accomplished computationally when the number of candidate predictors is arbitrary. 

We have presented four examples of how our framework can be applied. The first example involved selecting a cost-optimal method from a set of seventeen pathogenicity predictors. The results demonstrated how the clinical context could affect the preferred method (Figure 4C). Moreover, the findings highlighted that there is no single optimal method that can be applied across the entire clinical space, which contrasts with the view presented by AUC (Figure 4D) or MCC (Supplementary Figure S1B). These results underscore the importance of using measures that integrate properties of the deployment context instead of relying solely on predictive performance measures when choosing pathogenicity predictors for real-world applications. However, it is crucial to note that our findings do not suggest the universal superiority of one measure over the other. Instead, we believe that both predictive performance and cost-based measures complement each other, as they hold value at different stages of the development and application process of pathogenicity predictors. Metrics such as AUC or MCC are well-suited for evaluating progress in solving the scientific classification problem [38]. In such cases, the clinical context is irrelevant, as the ultimate goal remains consistent: distinguishing between pathogenic and benign variants based on scientific principles (biophysics, biochemistry, etc.) independent of specific contexts. In such cases, the clinical context is irrelevant since the goal, distinguishing between pathogenic and benign variants, is always the same and its solution depends on universal scientific principles (biophysics, biochemistry, etc.). Nevertheless, once the predictors have been developed and their clinical application is being considered, the situation undergoes a significant shift. In a clinical setting, every decision has a cost for stakeholders [9], which depends on the specific context. Consequently, cost emerges as a natural metric for assessing and comparing pathogenicity predictors. Our results shed light on the cost-based aspects of these tools, aiding developers in identifying aspects of their predictors’ performance that require improvement to enhance competitiveness.

Another important finding emerged from the comparison of MISC (Figure 2A) and MISC+REJ (Figure 4C): taking into account the rejection rate in the comparison of predictors had a significant impact on the final outcome. Treating pathogenicity predictors as classifiers with no reject option can lead to suboptimal decisions in many clinical scenarios.

Our second application of the cost framework involved analyzing the *TP53*-adapted guidelines for the clinical interpretation of sequence variants [34]. The results of this analysis (Figure 6) align with our previous findings, indicating that (i) there is no single optimal predictor that can be applied across the entire clinical space, and (ii) taking into account the rejection rate has a substantial impact on the selection of predictors. These results open the way to improve expert recommendations, making them more aware of the existing national and international differences between clinical settings. Advancing in this direction will require an effort on the part of the evaluation panels to find sets of cost parameters (*rc*_0_, *rc*_1_, and *rc*_2_) representative of different scenarios. While it may be challenging to find exact values, working with the ratios of these parameters is a feasible alternative since they are easier to estimate by experts [8].

In our third and fourth applications, we compared predictive methodologies aimed at clinical variant interpretation within the context of the ACMG/AMP guidelines [5]. One of the comparisons involved a comprehensive set of tools, precisely thirteen predictors examined in a study by Pejaver et al. [33] for their value as a source of computational evidence in clinical variant interpretation. The other comparison focused on two distinct sources of computational evidence [15,32] interpreting missense variants in the *ATM* gene. In both cases, we observed (Figure 7) a consistent pattern similar to our previous findings: strategies for classifying variants as pathogenic or benign exhibit optimal performance only within a specific subset of the clinical space. The size of this subset is influenced by the procedure’s sensitivity, specificity, and rejection rate. Therefore, to adequately encompass the entire clinical space in cost-optimal terms, it may be necessary to consider more than one predictive approach.

When considering collectively the findings from the application studies presented (Section 2.3, Section 2.4, Section 2.5 and Section 2.6), valuable insights regarding the use of pathogenicity predictors in clinical contexts emerge. Notably, REVEL, whose clinical usage in the clinical context has received significant support from the work of Pejaver et al. [33], exhibits promise across various scenarios (Figure 2 and Figure 7C,D). However, the range of REVEL’s applicability is contingent upon the stringency of the associated rejection region. Our findings suggest that the rejection regions utilized for REVEL, both in the *ATM* case [32] and in Pejaver et al.’s work [33], constrain its general applicability to clinical scenarios where rejection costs are low (Figure 6 and Figure 7A,B). Conversely, BayesDel demonstrates strong performance in both the *TP53* analysis (Figure 6) and the analysis of Pejaver et al.’s data (Figure 7A,B), establishing it as a viable alternative to REVEL in the opposite case, where rejection costs are high.

Finally, we would like to mention that the methodology presented here is not limited to the specific problem of pathogenicity prediction of missense variants. On the contrary, it can be applied to compare any type of bioinformatics or machine learning predictors for which we can define sensitivity, specificity, and rejection rate. 

## 4. Methods and Materials

Section 4.1 and Section 4.2 depict the two versions of the cost framework (without and with rejection term) we have created for comparing multiple classifiers across clinical scenarios. Each section follows the same structure: we describe the corresponding cost model and then present the computational procedure for addressing the multiple comparison problem. The more complex mathematical proofs are provided in the Supplementary Materials, Appendix S1, and supported by Supplementary Figures S2–S8. Subsequent Section 4.3 and Section 4.4 describe the variant dataset and pathogenicity predictors employed.

### 4.1. Framework for Comparing Classifiers with No Reject Option 

#### 4.1.1. Cost Model for Misclassification Errors Only 

Here, we utilize the standard cost model that considers only misclassification errors [37] and does not include any term for rejection. We shall refer to this model as MISC for simplicity. While it is not appropriate for classifiers that include a rejection option, the straightforward nature of MISC allows us to introduce the fundamental concepts of cost-based comparison among multiple predictors across diverse scenarios.

In this framework, the average misclassification cost of using a pathogenicity predictor in a clinical scenario is expressed as [37]:

*c = ρ*(1 − *s_e_*)*c*_0_
*+* (1 − *ρ*)(1 − *s_p_*)*c*_1_ (1)

where *ρ* and 1 − *ρ* are the frequencies of the pathogenic and benign variants, respectively. *ρ* is comprised between 0 and 1 and varies with the genome region sequenced and the population of individuals tested. *c*_0_ and *c*_1_, are misclassification costs [37] and denote the cost of annotating pathogenic variants as benign and benign variants as pathogenic, respectively. These two parameters encapsulate the clinical context into the cost formalism, capturing the essential factors that are important to healthcare users of pathogenicity predictors, such as medical or economic concerns, and patient and patient family considerations [9]. The values of *c*_0_ and *c*_1_ will differ depending on the users and the factors they wish to include in their evaluations of risks and costs. Finally, *s_e_* and *s_p_* in (1) are the sensitivity and specificity of the pathogenicity predictor, respectively; they are estimated by testing the predictor in a set of *N_p_* pathogenic and *N_b_* benign variants as follows:

(2a) $$s_{e}=\frac{TP}{N_{p}}$$

(2b) $$s_{p}=\frac{TN}{N_{b}}$$

where *TP* (True Positive) and *TN* (True Negative) are the numbers of correctly predicted pathogenic and benign variants, respectively. 

Following the method set out by Hernández-Orallo et al. [39], we normalize *c* using *c_T_ = c*_0_ *+ c*_1_, the cost magnitude. We obtain a normalized average cost, *rc*:

(3) $$rc=\frac{c}{c_{T}}=\rho (1-s_{e}){rc}_{0}+(1-\rho )(1-s_{p}){rc}_{1}$$

where *rc*_0_ = *c*_0_/*c_T_* and *rc*_1_ = *c*_1_/*c_T_*, and *rc*_0_ + *rc*_1_ = 1.

Working with *rc* simplifies subsequent analyses because *c*_0_ and *c*_1_ have an indefinite variation range, while their normalized equivalents, *rc*_0_ and *rc*_1_, vary between 0 and 1. 

We simplify Equation (3) by replacing *rc*_0_ with 1 *− rc*_1_:

*rc* = [(1 − *ρ*)(1 − *s_p_*) − *ρ*(1 − *s_e_*)]*rc*_1_
*+ ρ*(1 − *s_e_*) (4)

In Equation (4), clinical scenarios are now represented by their *rc*_1_ values. The range of *rc*_1_ values, the interval ***I*** = (0,1), is that of all possible clinical scenarios, and we will call it ‘clinical space’.

#### 4.1.2. Predictor Comparison across Clinical Scenarios

Comparing predictors based on cost values is a simple process. Given a clinical scenario characterized by a *rc*_1_ value, we can compare any number *N* of predictors using *rc*. We just need to calculate the *rc* of each method using Equation (4), sort all the resulting *rc* values, and choose the method with the lowest *rc.* This method will have the least average misclassification costs when deployed.

The previous procedure provides the most effective pathogenicity predictor for a particular scenario. However, there is no guarantee that the chosen predictor will consistently have the lowest *rc* for all potential application scenarios. In the following, we aim to address this problem, expanding the selection procedure to all possible scenarios (all *rc*_1_ values). Specifically, we intend to divide *I*, the clinical space, into a set of sub-intervals where each interval has a different method with the lowest *rc*.

Our approach is based on the fact that we can interpret the cost Equation (4) for each predictor as that of a line in *rc*_1_. Comparing predictors based on cost is then analogous to identifying the *rc*_1_ value at which their lines intersect. This value will split ***I*** into two parts, each dominated by a single predictor. If the intersection value falls outside ***I***, then a single method will prevail throughout the interval. The generalization of this concept to *N* predictors is as follows.

For a set {*M_i_*, *i* = 1, *N*} of *N* predictors, the division of ***I*** cannot be determined manually or visually due to the complexity of the line pattern, especially when *N* exceeds 2–4 predictors. In order to obtain the optimal division of ***I***, a computational approach is necessary, which can be achieved by following the four steps below. These steps ensure that the resulting division assigns the most cost-efficient (lowest *rc*) predictor to each point in ***I***:

**Step 1.** Solve in *rc*_1_ all the equations *rc*(*M_i_*) *= rc*(*M_j_*), (1 *≤ i ≤ N−*1*; i < j ≤ N*). The set of solutions obtained is: {${rc}_{1,int}^{i,j}$*;* 1 *≤ i ≤ N−*1*, i < j ≤ N*}, where the indexes *i* and *j* refer to the *M_i_-M_j_* comparison.

**Step 2.** Eliminate from the set of solutions all the points falling outside***I***. Then, sort the remaining values: 0 *<* ${rc}_{1,int}^{i,j}$ *<* ${rc}_{1,int}^{k,r}$ *<…<* ${rc}_{1,int}^{s,t}$ *<* 1. Note that between two successive *rc*_1*,int*_ values, there is no pair of *rc* lines crossing each other.

**Step 3.** For each of the associated intervals (0, ${rc}_{1,int}^{i,j}$), (${rc}_{1,int}^{i,j}$, ${rc}_{1,int}^{k,r}$),…,(${rc}_{1,int}^{s,t}$, *1*), find the predictor with the lowest *rc* at the interval’s midpoint. This predictor will have the lowest *rc* value all over the chosen interval because, within intervals, *rc* lines do not cross each other (see Step 2).

**Step 4.** Unify those adjacent intervals for which the same predictor has the lowest *rc*, repeating this step until all adjacent intervals correspond to different methods. Because of this univocal correspondence between intervals and predictors, the resulting list of intervals, {***I****_i_* = (*a_i_*,*b_i_*)*;* 1 *≤ i ≤ m*} gives the desired distribution of predictors across***I***. Note: *m < N*, since not all the methods are necessarily present in the final list. 

By following these steps, we can ensure that the predictor assigned to each point in ***I*** is the most cost-efficient. Specifically, any point in ***I*** belongs to one of its sub-intervals, and the predictor assigned to that interval has the lowest cost among all predictors (Step 3).

Results shown in Section 2.2 present an example of the application of this methodology to a set of seventeen predictors. 

**Interpreting the solution**. The list of intervals ***I****_i_* and their associated predictors is the solution to the problem of comparing *N* predictors across the clinical space using the MISC model. A simplified, predictor-centered view of this solution can be obtained by calculating the size of each interval, which is equal to *|****I****_i_|= b_i_-a_i_*, for ***I****_i_* = (*a_i_*,*b_i_*). This value represents the fraction of clinical scenarios in which the predictor *M_i_* is the most cost-efficient choice among all the predictors. 

It is important to note that the list of intervals/predictors obtained depends on the value of *ρ*. This dependence is explored further in the application of this formalism to a set of seventeen chosen pathogenicity predictors (see Results Section 2.2).

A python implementation of this procedure is available at (accessed on 1 June 2023): https://github.com/ClinicalTranslationalBioinformatics/clinical_space_partition.

### 4.2. Framework for Comparing Classifiers with Reject Option

#### 4.2.1. Cost Model for Misclassification Errors plus Rejection

Here, our starting point is the cost model for classifiers with reject option in [14], which we extend by replacing the part corresponding to the misclassification error with the more general expression described in Equation (1). We shall refer to this model as MISC+REJ for simplicity. 

In this framework, the average misclassification and rejection cost of using a pathogenicity predictor in a clinical scenario is expressed as [14]:

*c = αρ*(1 − *s_e_*)*c*_0_
*+ α*(1 − *ρ*)(1 − *s_p_*)*c*_1_*+* (1 − *α*)*c*_2_ (5)

In Equation (5), the parameters *s_e_*, *s_p_*, *ρ*, *c*_0_, and *c*_1_, are the same as in Equation (1). There are two new parameters: *c*_2_, the cost associated with rejection; *α*, the coverage of the predictor. The latter is directly related to the rejection rate, which is equal to (1 − *α*). *α* is computed as:

(6) $$\alpha =\frac{N}{N_{tot}}$$

where *N* is the number of cases from a total of *N_tot_* variants (a mixture of pathogenic and benign cases) for which the predictor generates a result. It should be noted that the observations made about *c*_0_ and *c*_1_ in the explanation of Equation (1) also apply here and include *c*_2_.

As before, instead of *c* we will use *rc*, the normalized average cost, obtained after dividing both sides of Equation (5) by *c_T_* (*=c*_0_ *+ c*_1_ *+ c*_2_):

(7) $$rc=\frac{c}{c_{T}}=\alpha \rho (1-s_{e}){rc}_{0}+\alpha (1-\rho )(1-s_{p}){rc}_{1}+(1-\alpha ){rc}_{2}$$

where *rc_i_ = c_i_/c_T_* (*i* = 0, 2) are comprised between 0 and 1, and *rc*_0_ *+ rc*_1_ *+ rc*_2_ = 1.

We reduce the number of parameters in *rc* by replacing *rc*_2_ with 1 − *rc*_0_ − *rc*_1_ in (7):

*rc* = [*αρ*(1 − *s_e_*) *+ α* − 1]*rc*_0_
*+* [*α*(1 − *ρ*)(1 − *s_p_*) *+ α* − 1]*rc*_1_
*+* 1 − *α* (8)

*rc* is now defined over a triangular region ***T*** in the *rc*_0_ − *rc*_1_ plane, bounded by the axes *rc*_0_, *rc*_1_, and the line *rc*_0_ *+ rc*_1_ = 1. ***T*** is conceptually equivalent to ***I*** in the MISC case: each point in ***T*** corresponds to a clinical scenario. We will also refer to ***T*** as ‘clinical space’. However, ***I*** and ***T*** differ in that the second is two-dimensional, i.e., clinical scenarios are represented by (*rc*_0_, *rc*_1_) pairs, not by a single value. 

#### 4.2.2. Predictor Comparison across Clinical Scenarios

Comparing any number *N* of predictors within a clinical scenario specified by a pair of values (*rc*_0_, *rc*_1_) is a matter of calculating (Equation (8)) and sorting their *rc* values. The most cost-optimal predictor would be the one with the lowest *rc* value. However, extending this procedure to all possible clinical scenarios is more complex than in the MISC case because we now have two parameters (*rc*_0_ and *rc*_1_) instead of just one. This means that we need to partition a two-dimensional space, rather than a one-dimensional interval, into a set of *m* regions {*r_k_*, *k* = 1, *m*}, such that each region corresponds to a single method that is the most cost-optimal within that region. To explain how we obtain these regions, we will first describe the case of two predictors (*N* = 2), and then extend this approach to an arbitrary number of predictors. A more detailed description of the methodology is provided in the Supplementary Materials, Appendix S1, where we also prove the most relevant results.

Let *M_i_* and *M_j_* be two pathogenicity predictors, and *rc*(*M_i_*) and *rc*(*M_j_*) their respective *rc*’s. We seek a division of ***T*** into two regions: *r_i_*, where *M_i_* is preferable to *M_j_* (*rc*(*M_i_*) *< rc*(*M_j_*)), and *r_j_*, where the opposite is the case (*rc*(*M_i_*) *> rc*(*M_j_*)). The boundary between *r_i_* and *r_j_* is defined by the equation *rc*(*M_i_*) *= rc*(*M_j_*), which, using Equation (8) for *rc*(*M_i_*) and *rc*(*M_j_*), gives:

{*ρ*[*α_i_*(1 − *s_e,i_*) − *α_j_*(1 − *s_e,j_*)] *+ α_i_* − *α_j_*}*rc*_0_
*+* {(1 − *ρ*)[*α_i_*(1 − *s_p,i_*)*-α_j_*(1 − *s_p,j_*)] *+ α_i_* − *α_j_*}*rc*_1_
*+ α_j_* − *α_i_* = 0 (9)

where *s_e,k_*, *s_p,k_,* and *α_k_* are the sensitivity, specificity, and coverage of predictor *M_k_* (*k = i*, *j*). Equation (9) shows that the boundary sought is a line, which we will call *l_ij_*, in the *rc*_0_*-rc*_1_ plane.

When *l_ij_* crosses ***T***, it divides it into two convex polygons, corresponding to the *r_i_* and *r_j_* regions. If *l_ij_* does not cross ***T***, then only one of the two methods will have the lowest *rc* in all ***T*** points. 

From Equation (9), we see that *l_ij_* depends on *ρ*; consequently, different values of this parameter may change *r_i_* and *r_j_*. This dependence is explored further in the application of this formalism to a set of seventeen chosen pathogenicity predictors (Supplementary Table S1).

For more than 2–3 methods, dividing ***T*** manually/visually becomes unfeasible. Here, we present a computational procedure for comparing an arbitrary number of predictors and find the {*r_k_*, *k* = 1, *m*} regions. Our approach is based on the following results. Firstly, all possible pair comparisons between predictors give rise to lines that divide ***T*** into a set of convex polygons (*P_N_*), within each of which a single predictor prevails. The proof of this result can be found in the Supplementary Materials (Appendix S1, Section S2). Secondly, grouping these polygons according to their associated methods yields the desired regions. Lastly, these polygons can be obtained using an adapted version of the Breadth First Search algorithm. Further details on the last two points are provided below.

The results shown in Section 2.3 present an example of the application of this methodology to a set of seventeen predictors. 

##### Finding the {*r_k_*, *k* = 1, *m*} Regions from the Polygons in *P_N_*

To obtain the regions *r_k_*, we use the polygons in *P_N_* as follows. Firstly, we determine the predictor with the lowest *rc* within each polygon. To accomplish this, we perform the following steps: (i) compute the average of the polygon’s vertices, (ii) calculate the *rc* value for each predictor at this average point, and (iii) sort the resulting *rc* values to select the method with the lowest *rc*. This method is then associated with the polygon. After this procedure, we have a list of polygons and their associated methods. Secondly, to obtain the desired regions *r_k_*, we simply merge the polygons that correspond to the same predictor. For instance, if there are three polygons in *P_N_* associated with the predictor *M_i_*, merging them will yield a region that is associated with *M_i_*. It is important to note that *m*, the number of regions, may not be equal to the number of predictors.

##### Using an Adapted Breadth First Search (BFS) to Generate Polygons in *P_N_*

We need to identify the polygons in *P_N_* to determine the {*r_k_*, *k* = 1, *m*} regions. To obtain these polygons, we first need to find their vertices, which are the intersection points between the lines *l_ij_* and between these lines and the sides of the triangle. Once the vertices are found, we can loop over them, enumerating the polygons meeting at each vertex. We can model this part as a cycle enumeration problem in graph theory.

Our starting point is the unweighted, undirected graph *G*(*V*, *E*), whose sets of vertices, *V*, and edges, *E*, correspond to *VP* and *EP*, the sets of vertices and edges of the polygons, respectively. Because the list of vertices of a polygon is formally equivalent to that of a cycle, we can reformulate the original looping through *VP* elements as a looping through *V* elements. For each *v_i_* ∈ *V*, we will use BFS as a shortest cycle generator. Because, in some cases, the resulting cycles correspond to figures with unwanted geometrical properties, we will keep only those cases that meet seven conditions (C1–C7, see Supplementary Material, Appendix S1, Section S3) designed to ensure that the associated figures correspond to polygons in *P_N_*. It can be shown that the exhaustive application of BFS under C1–C7, when looping through the vertices in *G*(*V*, *E*), produces the list of polygons in *P_N_*. The proofs of all the lemmas and propositions behind this procedure are given in the Supplementary Material (Appendix S1, Section S3). 

**Interpreting the solution**. The solution to the challenge of comparing *N* predictors in clinical space using the MISC+REJ model is a list of *r_k_* regions and their associated predictors. A simplified, predictor-centered view of this solution can be obtained computing the surface area of each *r_k_* region. This number represents the fraction of clinical scenarios where the predictor linked to *r_k_* is more preferable than the other predictors, in terms of *rc*. 

The dependence of these results on *ρ* is explored further in the application of this formalism to a set of seventeen chosen pathogenicity predictors (see Results Section 2.3).

A python implementation of this procedure is available at: https://github.com/ClinicalTranslationalBioinformatics/clinical_space_partition (accessed on 1 June 2023).

This code can recreate the results of the study and permits users to evaluate different predictor combinations. Nonetheless, when working with large sets of predictors, it is advisable to divide them into smaller groups and execute the program separately for each set. This is to avoid numerical errors that may occur during geometric computations [40], especially when dealing with low *ρ* values. The surviving predictors from these individual runs can be merged, and the process can be repeated until the remaining predictors can be managed in one run, i.e., when there are between 5 and 10 predictors left.

### 4.3. Variant Dataset 

For each predictor, we estimated the three performance parameters used for cost models, sensitivity (*s_e_*), specificity (*s_p_*), and coverage (*α*), in a set of benign and pathogenic variants retrieved from the database for nonsynonymous SNPs’ functional predictions (dbNSFP) database [41]. This database offers precomputed pathogenicity predictions for all potential nonsynonymous and splice-site single nucleotide variants in the human genome. The database’s latest version incorporates 36 deleteriousness prediction scores. For this work, we have used version 4.0a, release: 3 May 2019. We chose this version because it was released after the publication dates of the seventeen predictors used in this work (see next section). This helped us prevent the effect of first-order circularities [42] in estimating sensitivities, specificities, and coverages. There is only one exception to this rule: the predictor EVE [29], published in 2021. However, because this method is unsupervised, it is immune to circularity problems. 

We imposed three filters on the variants retrieved using information from the ClinVar database [43]. The database contains clinical significance interpretations for germline and somatic variants of any size, type, or genomic location linked to a range of conditions. It has five classes describing variants’ clinical significance: ‘Benign’, ‘Likely benign’, ‘Likely pathogenic’, ‘Pathogenic’, and ‘Uncertain significance’. The latter were not used in this work. We have utilized this information to create a curated collection of missense variants. Our approach excluded variants affecting splicing sites and included only variants with the review status of ‘Practice guideline’, ‘Expert Panel’, or ‘Criteria provided, multiple submitters, no conflicts’, and unifying clinical significance classes. Specifically, we combined ‘Benign’ and ‘Likely benign’ variants into the ‘benign’ class, and ‘Pathogenic’ and ‘Likely pathogenic’ variants into the ‘pathogenic’ class. The resulting dataset comprised 1902 variants, 809 pathogenic and 1093 benign, from 903 proteins.

### 4.4. Pathogenicity Predictors

We have demonstrated the utilization of our framework for selecting cost-optimal pathogenicity predictors by utilizing a collection of seventeen pathogenicity predictors selected using a qualitative combination of three criteria. Firstly, the predictor set needed to encompass various values of the three performance parameters utilized in this study: sensitivity, specificity, and coverage. Secondly, preference was given to the methods that were significantly cited in the literature [44] or recommended in adapted versions of the guidelines for clinical variant interpretation [5]. Lastly, the technical range of the methods had to be approximately representative of the whole set of methods. Below we briefly describe the chosen pathogenicity predictors.

CADD (**C**ombined **A**nnotation **D**ependent **D**epletion) [18]. CADD is a widely used tool for assessing genetic variant deleteriousness. It is a machine learning model utilizing over 60 features to score variants and prioritize causal variants for severe Mendelian disorders. 

EVE (**E**volutionary model of **V**ariant **E**ffect) [29]. This recently published method uses deep generative models to predict variant pathogenicity by analyzing the distribution of sequence variation across organisms. Based on an unsupervised machine learning approach, the results obtained are particularly promising.

LRT (**L**ikelihood **R**atio **T**est) [22]. It uses a DNA sequence evolutionary model that can accurately identify deleterious mutations that disrupt highly conserved amino acids in protein-coding sequences, likely causing disease.

MetaLR [24], MetaSVM [24]. These two related predictors are based on two ensemble scores that integrate the results of pre-existing tools using LR and SVM algorithms, respectively.

MutationAssessor [20]. Its score predicts the functional impact of amino acid replacements using a combinatorial entropy measurement applied to the multiple sequence alignment of the protein carrying the variant. It has been validated on a large set of variants and is useful for assessing mutations in cancer and missense variants.

MutPred [26]. It is a pathogenicity predictor with an associated probabilistic model that allows users to create a mechanistic view of the impact of genetic variants on protein structure and function.

MutationTaster2 [19]. It is a web-based tool that predicts the pathogenic potential of DNA sequence alterations, including the amino acid substitutions, intronic and synonymous alterations, indel mutations, and variants spanning intron-exon borders.

PMut [30]. Combines a variety of predictive features, from amino acid indexes to different measures of sequence conservation obtained from multiple sequence alignments. Predictions are available through a website where multiple queries are also possible.

PolyPhen2-HDIV and PolyPhen2-HVAR [16]. These are two versions of the predictor PolyPhen (**poly**morphism **phen**otyping). It combines sequence- and structure-based information to predict the functional impact of variants. PolyPhen2 has been broadly used in biomedical applications [44] and it is amply used in the development of metapredictors [4]. 

PON-P2 [27]. A Random Forest-based predictor that uses eight predictive features to classify missense variants. It has a good success rate although its rejection rate is high, resulting in a low coverage that may limit its applicability in some clinical settings.

PROVEAN (**Pro**tein **V**ariation **E**ffect **An**alyzer) [23]. PROVEAN predicts the impact of amino acid substitutions or indels on a protein’s biological function using a score related to pairwise alignment scores.

REVEL (**R**are **E**xome **V**ariant **E**nsemble **L**earner) [21]. REVEL is a method for predicting the pathogenicity of rare coding variants. It combines the scores of pre-existing predictors, resulting in a highly competitive tool that outperforms individual tools and other ensemble methods. It has been recently identified as a highly reliable source of computational evidence for clinical diagnostics [33].

SIFT (**S**orting **I**ntolerant **f**rom **T**olerant) [17]. This sequence-based method is based on the use of multiple sequence alignments to identify disruptions in the conservation pattern that can be related to disease. Similar to PolyPhen2, SIFT has also been broadly used in biomedical applications [44] and it is frequently used in the development of meta-predictors [4].

SNAP2 (**S**creening for **N**on-**a**cceptable **P**olymorphisms) [28]. In this pathogenicity predictor, evolutionary information is combined with other sequence-based properties. Specifically, use is made of bioinformatics predictions of structure properties, like secondary structure or accessibility.

VEST4 (**V**ariant **E**ffect **S**coring **T**ool) [25]. It relies on a Random Forest classifier for its predictions to which it assigns *p*-values incorporating a statistical hypothesis testing framework. It is regularly used in biomedical studies [44], like the adapted guidelines for the interpretation of variants in the *ATM* gene [15]. 

For each variant in our dataset, we retrieved the pathogenicity prediction of these tools from the dbNSFP [45] database, except for PON-P2, SNAP2, and PMut, for which we used the corresponding website. 

## 5. Conclusions

To assist in selecting the most suitable pathogenicity predictor taking into account clinical context, we have developed a comprehensive cost framework comprising formalism and computer code (referred to as MISC+REJ). Within this framework, pathogenicity predictors are treated as classifiers with a rejection option. We applied this model to four distinct examples, highlighting how clinical settings impact predictor preferences. In the first example, we utilized a set of seventeen pathogenicity predictors to emphasize the importance of incorporating rejection rates when comparing such predictors. We compared the outcomes of the MISC+REJ model with those generated by a simpler cost model (MISC), which lacks a rejection term. The results underscored the significance of including rejection rates in the evaluation process. Next, we employed the MISC+REJ cost framework to examine the *TP53*-adapted guidelines for variant interpretation. This analysis revealed that the optimal pathogenicity predictors can vary depending on specific clinical contexts. The findings demonstrated the necessity of considering context-specific factors when selecting predictors. The third and fourth examples involved the use of cost models to analyze the computational evidence utilized in clinical guidelines. Once again, we observed a consistent trend: achieving cost-optimal coverage of the clinical space requires employing multiple predictive approaches. Consequently, relying on a single method may result in suboptimal decisions within certain clinical settings.

## Data Availability

Performance data of the pathogenicity predictors mentioned are available in the Supplementary Tables S1–S3.

## Supplementary Materials

The supporting information can be downloaded at: https://www.mdpi.com/article/10.3390/ijms241411872/s1. References [46,47,48,49] are cited in the supplementary materials.
