# Supplementary material for: Choosing Variant Interpretation Tools for Clinical Applications: Context Matters
Source: Int J Mol Sci. 2023 Jul 24;24(14):11872. doi: 10.3390/ijms241411872 (PMC10380979; doi:10.3390/ijms241411872)
Supplement: Supplementary file 1 [file ijms-24-11872-s001.zip › ijms-2470538-supplementary.pdf]

SUPPLEMENTARY MATERIALS

Choosing Variant Interpretation Tools for Clinical Applications: Context Matters

Josu Aguirre [1,†], Natàlia Padilla [1,†], Selen Özkan [1], Casandra Riera [1], Lídia Feliubadaló [2,3] and Xavier de la Cruz [1,4,*]

1 Research Unit in Clinical and Translational Bioinformatics, Vall d'Hebron Institute of Research (VHIR), Universitat Autònoma de Barcelona, P/Vall d'Hebron, 119-129, 08035 Barcelona, Spain; aguirre.gomez.josu@gmail.com (J.A.); natalia.padilla@vhir.org (N.P.); selen.ozkan@vhir.org (S.Ö.);
mcasandrariera@gmail.com (C.R.)

2 Hereditary Cancer Program, Program in Molecular Mechanisms and Experimental Therapy in Oncology (Oncobell), IDIBELL, Catalan Institute of Oncology, 08908 L'Hospitalet de Llobregat, Spain;
lfeliubadalo@iconcologia.net

3 Centro de Investigación Biomédica en Red de Cáncer (CIBERONC), 28929 Madrid, Spain

4 Institució Catalana de Recerca i Estudis Avançats (ICREA), 08010 Barcelona, Spain

* Correspondence: xavier.delacruz@vhir.org; Tel.: +34-934-893-000 (ext. 2687)

† These authors contributed equally to this work.

Appendix S1. Description of the MISC+REJ cost model and proof of the main results underlying the clinical space search algorithm under this model

In this work, we present a framework to simultaneously compare the performance of multiple pathogenicity predictors in multiple deployment scenarios. The goal is to find which predictor is more appropriate for each scenario, or if a single predictor is enough for all scenarios. We treat pathogenicity predictors as classifiers with reject option because they have an incomplete coverage [1]. Our framework consists of two components. The first component is a theoretical body that includes a cost model and a set of propositions forming the foundation for the second component, the computational part. This computational part presents a procedure that divides the clinical space into regions where a single predictor prevails over the others in terms of cost. Here, we present the cost model, and in Sections S1-S3, we explain the computational procedure and the theoretical outcomes that support it.

NOTE. In the main body of the article, for comparison purposes, we present a framework based on the split misclassification errors (MISC) only; the reject term is not included. All the results about MISC are provided in the article.

TERMINOLOGY NOTE. We will use the terms 'classifier' and 'predictor' interchangeably. We will also refer to these tools as 'methods', particularly in the propositions, to underline the generalizability of the results.

The cost model we use as a starting point is the one used to evaluate classifiers with a reject option [2,3], which corresponds to the case of pathogenicity predictors. However, this model must be adapted to healthcare applications because of two reasons. First, it treats equally misclassification errors due to false positives and false negatives, and this is problematic because the medical consequences of these errors may vary substantially [4]. And second, it should take into account the frequency of the predicted classes [5–7], i.e., the frequency of

pathogenic and benign variants, in our case. In the following, we present a version of the cost model for reject classifiers (to which we will refer as MISC+REJ) based on these considerations (see the underlying probabilistic tree diagram in Supplementary Figure S2). In particular, the misclassification error is split into two terms, corresponding to false positive and false negative errors, and a parameter for the class probability. The cost model is then written as:

$$c = \alpha\rho(1-s_e)c_0 + \alpha(1-\rho)(1-s_p)c_1 + (1-\alpha)c_2 \qquad \text{(SA1)}$$

where the parameters c_0 and c_1 are the misclassification costs: c_0 is the cost associated to annotating pathogenic variants as benign, and c_1 is the cost associated to annotating benign variants as pathogenic. c_2 is the cost associated to prediction rejection. In general cost models, ρ and $1-\rho$ are the probabilities of the two predicted classes. In our case, ρ will be the frequency of pathogenic variants expected in the deployment context. The value of ρ, comprised between 0 and 1, varies with the genome region sequenced (gene panel, whole exome, etc.) and with the population of individuals tested (e.g., the individuals attending a specific hospital unit, ethnic group, etc.). s_e and s_p are the sensitivity and specificity of the pathogenicity predictor; α is its coverage (1-α is the rejection rate). These parameters are estimated testing the predictor in a set of N_{tot} variants as follows:

$$s_e = \frac{TP}{N_p} \qquad \text{(SA1.1)}$$

$$s_p = \frac{TN}{N_b} \qquad \text{(SA1.2)}$$

$$\alpha = \frac{N}{N_{tot}} \qquad \text{(SA1.3)}$$

where N ($N \leq N_{tot}$) is the total number of predicted variants; N_p, and N_b are the numbers of predicted pathogenic and benign variants. We have that: $N=N_p+N_b$. And $N_{tot}-N$ is the number of rejected predictions.

Following Hernández-Orallo et al. [7], instead of c we will use rc, the normalized average cost, which is obtained after dividing both sides of equation (1) by c_T ($=c_0+c_1+c_2$):

$$rc = \frac{c}{c_T} = \alpha\rho(1-s_e)rc_0 + \alpha(1-\rho)(1-s_p)rc_1 + (1-\alpha)rc_2 \qquad \text{(SA2)}$$

where $rc_i=c_i/c_T$ (i=0,2) are comprised between 0 and 1, and $rc_0+rc_1+rc_2=1$.

We can reduce the number of parameters in rc replacing rc_2 by $1-rc_0-rc_1$ in equation (2). We obtain, after some reordering:

$$rc = [\alpha\rho(1-s_e)+\alpha-1]rc_0+[\alpha(1-\rho)(1-s_p)+\alpha-1]rc_1+1-\alpha \qquad \text{(SA3)}$$

rc is defined over a triangular region ***T*** in the rc_0-rc_1 plane, bounded by the axes rc_0, rc_1 and the line $rc_0+rc_1=1$ (Figure 3a). ***T*** is conceptually equivalent to the interval ***I***=(0,1) in the MISC case (see main body of the paper): each point in ***T*** corresponds to a clinical scenario. We will refer to ***T*** as 'clinical space' also. ***I*** and ***T*** differ in that the second is two-dimensional; i.e. clinical scenarios are represented by (rc_0, rc_1) pairs, not by a single value.

Section S1. Generalizing predictor comparison to all clinical scenarios

For N predictors, in a clinical scenario defined by a pair (rc_0, rc_1), the comparison is straightforward: we only have to compute and sort their respective rc values. The tool of choice will be the one with the lowest rc; it will be the predictor with the lowest MISC+REJ cost. Here, the goal is to solve the more complex problem of comparing predictors across the clinical

space. That is, we want to find a division of this space in regions within which a single predictor prevails in terms of cost. The problem is more difficult for MISC+REJ than for MISC because the space we want to partition is two-dimensional instead of one-dimensional. To illustrate the procedure, we first describe the case of two predictors (N=2) and then we extend the idea to an arbitrary number N of predictors.

Let M_i and M_j be two pathogenicity predictors, and $rc(M_i)$ and $rc(M_j)$ be their respective rc's. We seek a division of $\boldsymbol{T}$ into two regions: r_i, where M_i is preferable to M_j ($rc(M_i) < rc(M_j)$), and r_j, where the opposite is the case ($rc(M_i) > rc(M_j)$). The boundary between r_i and r_j is defined by the condition $rc(M_i)=rc(M_j)$, which, using equation (3) for $rc(M_i)$ and $rc(M_j)$, gives:

$$\{\rho[\alpha_i(1-s_{e,i})-\alpha_j(1-s_{e,j})]+\alpha_i-\alpha_j\}rc_0+\{(1-\rho)[\alpha_i(1-s_{p,i})-\alpha_j(1-s_{p,j})]+\alpha_i-\alpha_j\}rc_1+\alpha_j-\alpha_i = 0 \quad \text{(SA4)}$$

where $s_{e,k}$, $s_{p,k}$ and α_k are the sensitivity, specificity and coverage of predictor M_k (k=i,j). Equation (4) shows that the boundary sought is a line (Figure 3a), which we will call l_{ij}, in the rc_0-rc_1 plane.

When l_{ij} crosses $\boldsymbol{T}$**,** it divides it into two convex polygons (Figure 3a), corresponding to the r_i and r_j regions. If l_{ij} does not cross $\boldsymbol{T}$**,** then only one of the two methods will have the lowest rc in all $\boldsymbol{T}$ points.

From Equation (4), we see that l_{ij} depends on ρ; consequently, different values of this parameter may change r_i and r_j (Supplementary Figure S3). In this appendix we concentrate on the problem of dividing $\boldsymbol{T}$ when more than two predictors are available, keeping the value of ρ fixed. In the main body of the paper, we describe how different values of ρ affect the resulting division.

To generalize the comparison to more than two predictors (M_i, i=1,N; N>2), we will develop a procedure that divides $\boldsymbol{T}$ into m regions, $\{r_k$, k=1,m$\}$, such that only one method per region has the lowest rc. (Note that m ≤ N, since there may be predictors that are never better

than the others). The next two sections are devoted to provide some fundamental results underlying this procedure and then to describe it, first in geometric (Section S2) and then in computational (Section S3) terms.

Section S2. Dividing *T* into a set of regions $\{r_k$, k=1,m$\}$ in which only one predictor per region has the lowest *rc*

Note. In our proofs we use several results about convex polygons that can be easily found in the books of Lee [8] and of Yaglom and Botyanskii [9]. The most important ones are explicitly cited.

Here, we address, in geometric terms, the problem of finding the $\{r_k$, k=1,m$\}$ regions in which only one predictor per region has the lowest *rc*. The starting point is the set of N predictors that we want to compare. Previously, we have seen that there is a line associated to each pairwise comparison between predictors (Equation (4)). Therefore, after doing all the possible M=N.(N-1)/2 pairwise comparisons, we obtain a set $L_N=\{l_{ij}$, i=1,N-1 *and* j=i+1,N$\}$ of lines. A first important result (Proposition S1) is that these lines cut ***T***, producing a division of this triangle into a set P_N of convex polygons that will be key to finding the r_k regions.

Proposition S1. Let N∈ℕ be an arbitrary number of methods and let $L_N=\{l_{ij}$, i=1,N-1 and j=i+1,N$\}$ be the set of lines resulting from all the pairwise comparisons between these methods, using *rc*. These lines cut ***T*** into a set $P_N=\{p_l\}$ of convex polygons.

Proof. By induction.

<u>Base case</u>. For N=*2*, there is only one line in L_N, since there is only one comparison between two methods. When the line contains no interior point of ***T***, either because it does not intersect with ***T***, or because it is a supporting line of it, P_2 will have a single element, ***T***, which is convex

because it is a triangle. If the line contains at least a point interior to ***T***, then it will cut ***T*** in exactly two points [9]. The line segment uniting these two points is a chord of the polygon [8] and, by the 'Polygon Splitting Theorem' [8], divides ***T*** into two convex polygons.

Induction step. Here we show that if the proposition is true for N, then it is true for N+1. That is, we want to show that if L_N divides **T** into a set of convex polygons P_N, then L_{N+1} divides ***T*** into a new set of convex polygons that we will call P_{N+1}.

We know that the set of lines resulting from the comparison of N+1 methods, L_{N+1}, will contain the lines corresponding to the comparisons between the first N methods, L_N, and between these N methods and an additional (N+1)th method, $\{l_{i,N+1}\}_{i=1,N}$, that is:

$$L_{N+1} = L_N \, U \, \{l_{i,N+1}, \text{ i=1, N}\} \qquad \text{(SA5)}$$

Cutting ***T*** with the lines in L_{N+1} is equivalent to cutting it with the lines in L_N and then with those in $\{l_{i,N+1}, \text{ i=1, N}\}$ since order is irrelevant to the final result. Therefore, P_{N+1} will be the result of cutting the polygons in P_N with the lines in $\{l_{i,N+1}, \text{ i=1, N}\}$. When we cut P_N with the first line, $l_{1,N+1}$, we create a new division of ***T*** in which each of the polygons split by $l_{1,N+1}$ will be replaced by two children polygons (i.e., no polygon traversed by a line remains in the new division of ***T***). Next, we will repeat this process for the remaining lines in $\{l_{i,N+1}, \text{ i=1, } N\}$ until we obtain P_{N+1}. At the end of each step, the division of ***T*** will be constituted by the set of $\mathrm{P_N}$ polygons unaffected by the $l_{i,N+1}$ line (these polygons are convex because the proposition is true for N), and by the children of the affected polygons. Given that the affected polygons are convex (again because the proposition is true for N), the children will also be convex, by the 'Polygon Splitting Theorem' [8]. Therefore, at the end of each step, the resulting division of ***T*** will be constituted by a set of convex polygons and, consequently, P_{N+1}, which is obtained at the end of the final step, will be constituted by convex polygons only. QED.

The polygons in P_N have several characteristics that are relevant for the computational algorithm used to list them (described in Section S3.3). First, their edges are noncollinear line segments that belong either to the L_N lines or to the three segments defining ***T.*** Second, their vertices can be: the ***T*** vertices, the intersection points between the L_N lines, and the intersection points between these lines and the triangle edges. Third, for each L_N line, the segment delimited by the intersection points of the line with the triangle is formed by a concatenation of edges from P_N polygons (Supplementary Figure S4). And fourth, the same happens for the three segments defining ***T,*** which are formed by a concatenation of edges from P_N polygons.

The P_N polygons also satisfy the following lemma.

Lemma S1. Let p be a polygon from P_N. Then none of its interior points belong to another polygon $q \in P_N$.

Proof. By contradiction. Let us assume that there exists a polygon $p \in P_N$ such that one of its interior points belongs to $q \in P_N$. This point will belong to one of the edges of q. The line from L_N containing this edge will cut p at two points [9], that is, it will traverse p. This is in contradiction with the procedure utilized to generate P_N, in which any polygon traversed by a line from L_N is removed from the polygon list and replaced by the two children polygons. Therefore, p does not exist. QED.

Finally, we will show a key property of the P_N polygons, used to build the regions r_k.

Proposition S2. Let P_N be the set of convex polygons obtained after dividing ***T*** using L_N, the set of lines associated to the pair comparisons between N methods. For each polygon $p \in P_N$, the lowest *rc* value at all its interior points always corresponds to the same method.

Proof. By contradiction. Let us assume that the proposition is not true. That is, that there exists a polygon $p \in P_N$ with an interior point m such that M_i, the method with the lowest *rc* value at m, is different from M_j, the method with the lowest *rc* value at the remaining interior points of p. Let us consider n, one of these remaining interior points. Then, according to our assumption, the *rc* values of M_i and M_j at m ($rc_m(M_i)$ and $rc_m(M_j)$, respectively) and at n ($rc_n(M_i)$ and $rc_n(M_j)$, respectively) satisfy the following inequalities: $rc_m(M_i)<rc_m(M_j)$ and $rc_n(M_i)>rc_n(M_j)$, respectively. This is in contradiction with the fact that the boundary line between M_i and M_j does not pass between m and n, because during the construction of P_N (Proposition S1) any polygon traversed by a line from L_N is removed from P_N and replaced by the resulting children polygons. QED.

We will say that a method M_k is associated to a polygon p, when M_k has the lowest *rc* value for the interior of p. Note, that M_k will also have the lowest *rc* value at the line segments defining p.

The polygons in P_N do not necessarily coincide with the r_k regions but can be used to obtain them using the following procedure.

Step 1. **Find the method associated to each polygon.** For each polygon in P_N we apply the next three steps:

Step 1.1. Compute the average of its vertices, which is a point belonging to the interior of the polygon because the polygon is convex.

Step 1.2. Compute the *rc* value, at this average point, for each of the N methods.

Step 1.3. Sort the N methods according to their *rc*'s at the average point and choose the method with the lowest *rc* value. By Proposition S2, this method prevails (has the lowest *rc*) at all the points interior to the polygon considered. This will be the method associated to the polygon.

Step 2. Obtaining the {r_k, k=1,m} regions. Each region r_k (Supplementary Figure S5) is obtained as the union of all polygons associated to the same method, M_k:

$$r_k = \cup_{i \in \Omega_k} p_i \tag{SA6}$$

where Ω_k are the indexes of the p_i polygons in P_N for which M_k has the lowest *rc* value. We will say that M_k is the method associated to the region r_k.

By application of Proposition S2 to the polygons associated to M_k, we know that there is only one method associated to each r_k. By the same reason, we know that no other region contains a point for which M_k is the method with the lowest *rc*. That is, by construction, there are no two regions with the same associated method; therefore, {r_k, k=1,m} is the set we are looking for.

Note. For clarity purposes, in this work we do not explicitly treat the fact that, at the boundary between adjacent r_k regions, two methods have the same *rc* value. This fact has no impact on the results presented, neither in the finding of the r_k regions, nor in computing their surfaces, etc.

Section S3. Computational obtention of the P_N polygons using an adapted Breadth First Search (BFS)

As we have seen in the previous section, once we know the polygons in P_N it is trivial to obtain the r_k regions, using (6). Here, we describe how we can obtain these polygons computationally. In particular, we show that the problem of finding a P_N polygon, in terms of its constituting vertices, can be modeled as a graph problem, and solved with an adapted version of the BFS algorithm that we will call aBFS. A python implementation of this procedure, CSP-rej (Clinical Space Partition, rejection), is available at:

https://github.com/ClinicalTranslationalBioinformatics/clinical_space_partition

This code reproduces the results presented in this work and allows users to explore other combinations of predictors. It must be noted that, when planning comparisons of large numbers of predictors, it is preferable to partition the original set of predictors into smaller sets, and run the program separately for each set. Then, aggregate the surviving methods from these separate runs and execute the program again. This approach will reduce the risk of numerical exceptions that appear in geometric computations [10], particularly when working with low ρ values. It can be repeated as many times as desired.

Preliminary results: computing the set of edges and vertices of the P_N polygons

The first step in our approach is to compute the set of lines $L_N=\{l_{ij},$ i=1,N-1 *and* j=i+1,N$\}$, applying equation (4) to all possible comparisons between the N methods.

The next step is to build *VP* and *EP*, the sets of vertices and edges, respectively, of the P_N polygons. For *VP*, we first compute the intersection between the lines in L_N, keeping only the points falling inside ***T.*** These points are included in *VP*. Then, we compute the intersection between the lines in L_N and the boundaries of ***T.*** The resulting points are added to *VP*. Finally, we include in *VP* the three vertices of ***T.*** The resulting set of vertices is used to obtain *EP*, which is constituted by all the $\overline{v_i v_j}$ (v_i, $v_j \in VP$) line segments uniting two consecutive vertices

in an L_N line or in the lines defining ***T.*** Note that every element of *EP* corresponds to the edge of a P_N polygon, as shown in the next lemma.

Lemma S2. Any segment in *EP* is the edge of at least one polygon in P_N.

Proof. By contradiction. We assume that there exists a segment $\overline{v_i v_j} \in EP$ which is not the edge of any P_N polygon. In the following, we show that the possible options for $\overline{v_i v_j}$ lead to a contradiction.

By construction of *EP*, $\overline{v_i v_j}$ belongs either to one of the lines in L_N, or to one of the three lines defining ***T.*** In all cases, within ***T*** these lines are formed by a concatenation of edges from polygons in P_N (Supplementary Figure S4). Therefore, $\overline{v_i v_j}$ will overlap with some of these edges. Two situations are possible. It may happen that $\overline{v_i v_j}$ spreads over two or more edges. In this case, some of the vertices of these edges will be comprised between v_i and v_j, in contradiction with the fact that v_i and v_j are consecutive. A second possibility is that $\overline{v_i v_j} \subsetneq \overline{v_k v_l}$, where $\overline{v_k v_l}$ is one of the edges in the line. This in contradiction with the fact that v_k and v_l are consecutive. Consequently, $\overline{v_i v_j}$ must be an edge of a P_N polygon. QED.

Before describing how we obtain the P_N polygons, we need to prove a result about polygons sharing more than one edge that will be used to prove our computational procedure.

Proposition S3. Let $p \in P_N$ be a polygon with two edges $\overline{v_i v_j}$, $\overline{v_i v_k} \in EP$ forming a consecutive angle (Supplementary Figure S6). There exists no other convex polygon q, different from p, with $\overline{v_i v_j}$ and $\overline{v_i v_k}$ among its edges and the remaining edges belonging to *EP*, and such that none of its interior points belongs to another polygon in P_N.

Proof. By contradiction. We will assume that q exists and explore the different possibilities that arise, showing that they all lead to contradiction. In particular, we will focus our reasoning on the relative position of the points in p and q outside $\overline{v_i v_j}$ and $\overline{v_i v_k}$. There are three possibilities, considering that both p and q are convex.

If in q these points are all interior to p, then the edges of q joining v_j and v_k will be interior to p (Supplementary Figure 7a). Because, these edges belong to EP, they then necessarily belong to polygons in P_N (by Lemma S2). This is in contradiction with the fact that none of the interior points of p belong to another P_N polygon (by Lemma S1).

If all the points in p outside $\overline{v_i v_j}$ and $\overline{v_i v_k}$ are interior to q (Supplementary Figure S7b) then there are interior points of q will belonging to a polygon in P_N, because $p \in P_N$. This is in contradiction with the definition of q as having no interior points from P_N polygons.

Finally, we reach a similar contradiction when both p and q have interior points from each another (in this case, the points may come from full segments or fragments of segments) (Supplementary Figure S7c). QED.

Building P_N with a graph-based approach

The list of polygons in P_N can be obtained looping through all the vertices in VP, enumerating the polygons that meet at each vertex. Here, we show that we can model this polygon enumeration problem as a cycle enumeration problem in graph theory.

Our starting point is the unweighted, undirected graph $G(V, E)$, whose set of vertices, V, and edges, E, correspond to VP and EP, respectively. Because the list of vertices of a polygon is formally equivalent to that of a cycle, we can reformulate the original looping through VP elements as a looping through V elements. The lists of vertices of the P_N polygons meeting at a given vertex will now correspond to cycles in $G(V, E)$.

In this search, for each $v_i \in V$, we will use BFS as a shortest cycle generator, keeping only those cycles satisfying the following conditions:

- C1. A cycle cannot have more than one edge corresponding to a segment from the same line. This rule is applied to eliminate those sequences of edges produced by the BFS that do not correspond to convex polygons, according to the edge-line lemma [8]. Also, to avoid that edges from collinear segments are included.
- C2. A cycle cannot have repeated vertices, except the first and the last one, as in polygons all the vertices are different except the first and the last one [8].
- C3. Every edge in E has a counter that is decreased by one each time it is included in a cycle. Once the counter reaches zero, the edge is excluded from future searches. The starting value of the counter of each E edge depends on the location of its equivalent edge in EP. If the latter belongs to a side of the triangle, the counter will start at 1; otherwise (when it belongs to a line in L_N) it will start at 2. This condition guarantees that, for each edge in EP, we enumerate all the polygons sharing it, thus ensuring, together with C4, that our polygon enumeration procedure is exhaustive for P_N elements.
- C4. Every vertex in V has a counter that is decreased by one each time it is included in a cycle. Once the counter reaches zero, the vertex is excluded from future searches. The starting value of the counter of each V vertex depends on the environment of its equivalent vertex in VP. More precisely, it depends on the number and origin of EP edges that include the VP vertex (see Supplementary Figure S8). This condition guarantees that, for each vertex, we enumerate all the polygons sharing it, thus ensuring, together with C3, that our polygon enumeration procedure is exhaustive for P_N elements.

- C5. Once a minimal cycle is found, it is excluded from future searches. This condition is introduced to avoid repetitions in the final list of cycles.
- C6. For every minimal cycle found, we check that the corresponding polygon has no interior points corresponding to vertices in *VP*. This condition, together with C7, prevents the inclusion in the final list of cycles of convex polygons not belonging to P_N (see Lemma S3 and Proposition S4 below).
- C7. For every minimal cycle found, we compute all the possible chords between the vertices of the corresponding polygon. If any of these chords corresponds to an edge in *EP*, the polygon is discarded. This condition excludes cycles with a list of vertices in which more than two vertices from the same line are included, thus limiting the chosen cycles to those corresponding to convex polygons (all pairs of non-adjacent edges of the chosen polygon are semiparallel and, by Theorem 8.7 in Lee, the polygon is convex). Condition C7, together with C6, also prevents the inclusion, in the final list of cycles, of convex polygons not belonging to P_N (see Lemma S3 and Proposition S4 below).

As mentioned above, we call aBFS this combination of BFS and C1-C7 conditions. We will now establish (Lemma S3 and Proposition S4) that a cycle found with aBFS corresponds to a polygon in P_N.

Lemma S3. Let c_{SC} be a cycle found by aBFS, and constituted by the sequence of edges $\{v_i, v_{i+1}\} \in E$, where i=1,N and $v_1=v_{N+1}$. Then, p_{sc}, the corresponding sequence of segments $\overline{v_i v_{i+1}}$ from *EP*, is a convex polygon and has no interior points from any polygon in P_N.

Proof. c_{SC} is a cycle whose sequence of edges correspond to a sequence of segments, from *EP*, characteristic of a polygon [8]: it starts and ends at the same vertex, there are no collinear segments (condition C1), and it has not repeated vertices (condition C2) other than the first and last ones, which are equal.

p_{sc} is convex because of conditions C1 and C7.

p_{sc} has no interior points from any polygon in P_N because of conditions C6 and C7. QED.

Proposition S4. Let c_{SC} be a Shortest Cycle identified by aBFS. Then, p_{SC}, the polygon corresponding to this cycle, belongs to P_N.

Proof. First, we know from Lemma S3 that p_{sc} is a convex polygon with no interior points from any polygon in P_N. Now, let us select an arbitrary pair of adjacent edges from p_{sc}, $\overline{v_i v_j}$ and $\overline{v_i v_k}$. By construction of EP, $\overline{v_i v_j}$ and $\overline{v_i v_k}$ are edges of polygons in P_N (Lemma S2). Because no line from L_N passes between them (by conditions C6 and C7), $\overline{v_i v_j}$ and $\overline{v_i v_k}$ belong to the same polygon $p \in P_N$. From Proposition S3 we know that p is the only convex polygon with $\overline{v_i v_j}$ and $\overline{v_i v_k}$ among its edges and no interior points from any polygon in P_N, therefore, $p_{SC} = p$ and $p_{sc} \in P_N$. QED.

For each vertex v_i, aBFS will find the shortest cycles corresponding to all the $\overline{v_i v_j}$, $\overline{v_i v_k}$ pairs forming consecutive angles. This procedure will be repeated for all the vertices in $G(V, E)$, guaranteeing, through the use of counters (conditions C3 and C4 above), that the number of cycles found matches that of expected P_N polygons. By condition C5 and Proposition S4, we know that the shortest cycles found are unique and correspond to P_N polygons, respectively.

In summary, exhaustive application of aBFS to the vertices in E will produce the list of polygons in P_N. Each polygon will be defined by its list of vertices.

Supplementary Figures

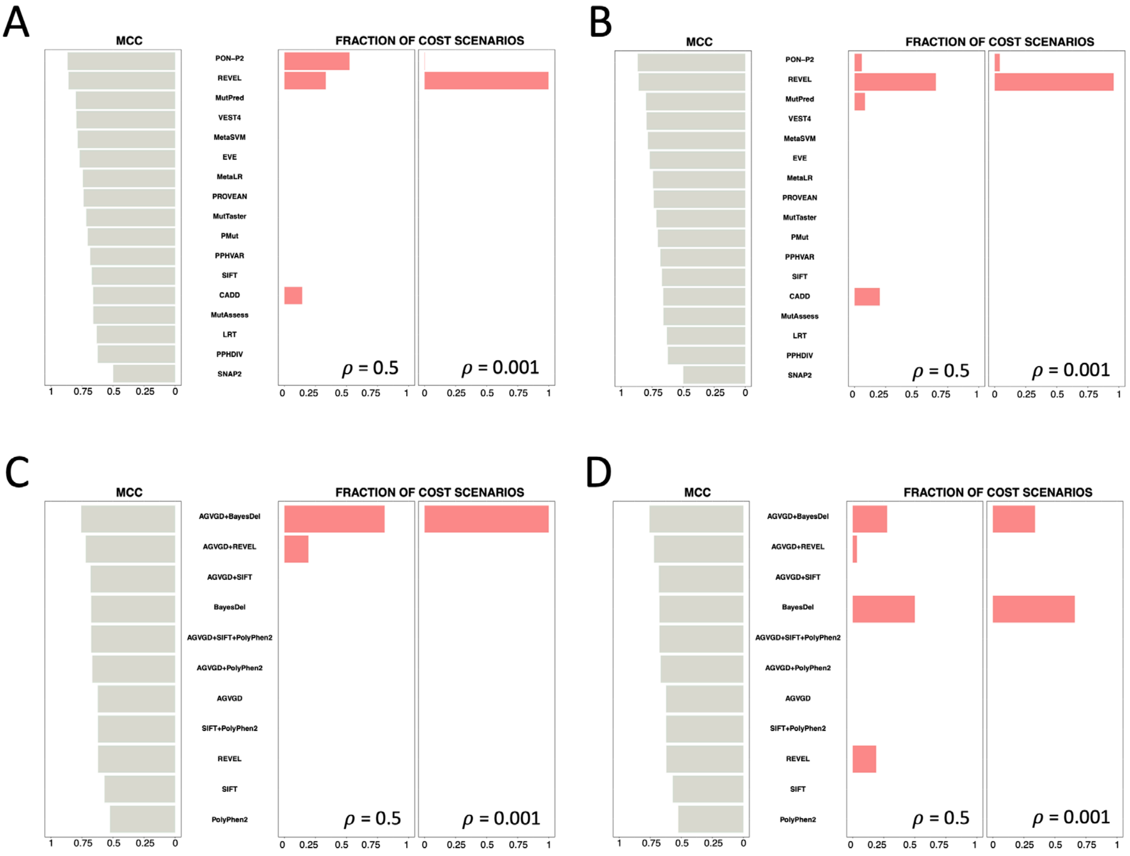

Supplementary Figure SA1. Comparison between the MCC ranking of the seventeen pathogenicity predictors and their corresponding fractions of cost scenarios. In all the parts of this figure (a-d), pathogenicity predictors are ranked according to their MCC (grey bars, left side) and fraction of cost scenarios for which each predictor is optimal are represented with pink bars, right side. The results are shown for ρ=0.5 and ρ=0.001). **A**, MISC analysis of the seventeen predictors. **B**, MISC+REJ analysis of the seventeen predictors. These figures are equivalent to those shown in Figs. 1b and 3d, respectively, for AUC. We have reproduced the MCC analysis using data for the TP53 gene and several predictors, retrieved from the work of Fortuno et al. [11]: **C**, MISC analysis, and **D**, MISC+REJ analysis. Here the cost space is

restricted to the Li-Fraumeni syndrome. The results confirm an incomplete correspondence between the MCC analysis and the fraction of cost scenarios in which each method predominates.

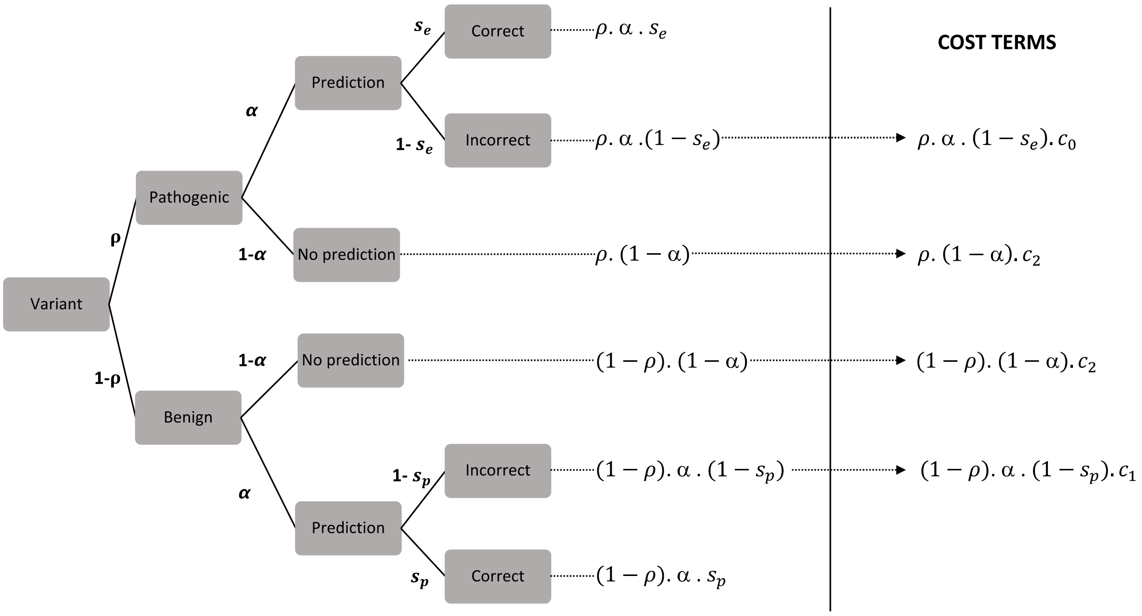

Supplementary Figure S2. Probabilistic tree diagram underlying the cost-framework presented in this work. Each branch of the tree corresponds to a different situation in the use of prediction methods, the situation's probability is written by its side. Multiplying probabilities along branches gives the probability of an event that is the combination of different situations. For example, a pathogenic variant can be incorrectly predicted as benign; the probability of this event is: $\rho.\alpha.(1\text{-}s_e)$. In the cost model MISC+REJ each of these events will contribute a term (shown to the right of the vertical line) that, after summation and reordering, will result in equation (1).

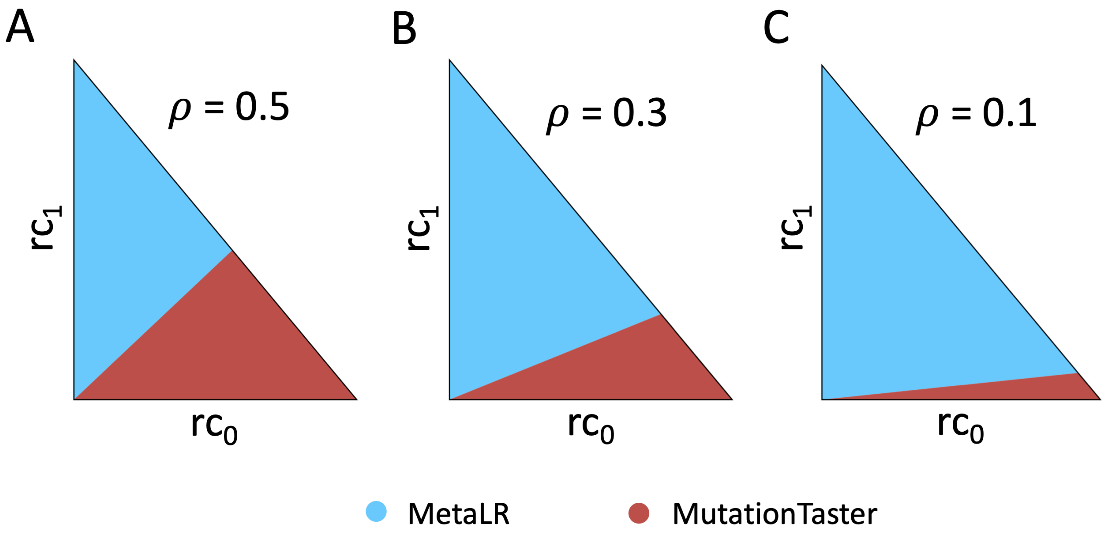

Supplementary Figure S3. Effect of the fraction of pathogenic variants in the sample (*ρ*) on the distribution of pathogenicity predictors over the cost domain. This figure shows how different values of *ρ* (***a***, *ρ* = 0.5; ***b***, *ρ* = 0.3; ***c***, *ρ* = 0.1) may substantially alter the size of the cost region assigned to each predictor. A version of this figure for the case of seventeen predictors is presented in Figure 4.

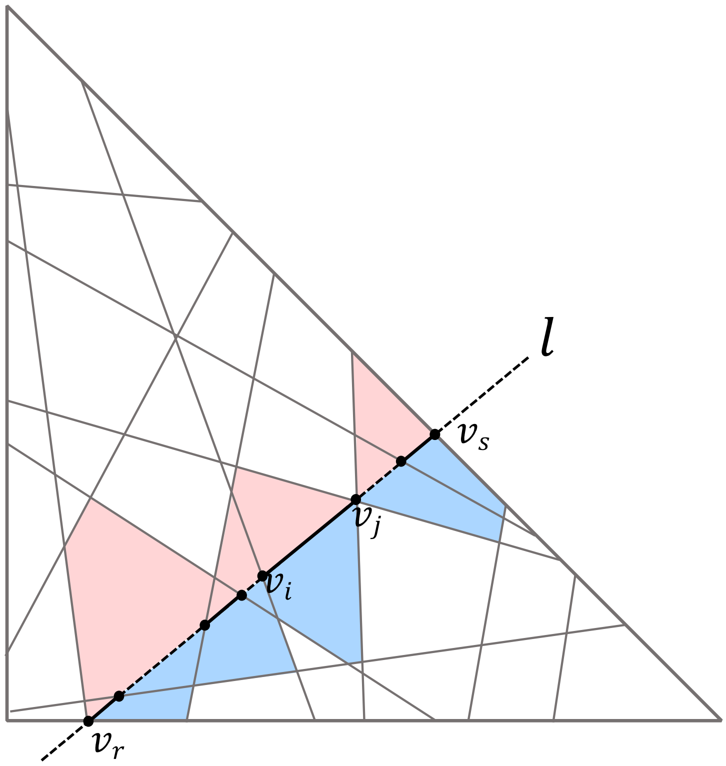

Supplementary Figure S4. Lines from L_N are constituted by a concatenation of edges from P_N polygons, when crossing the triangle *T*. The figure shows how a line $l \in L_N$ is formed by a succession of segments that correspond to edges of P_N polygons (pink/blue), when traversing the triangle ***T*,** i.e., between the points v_r and v_s. As illustrated for $\overline{v_i v_j}$, each segment is shared by two polygons, one above (pink) and the other below (blue) the segment.

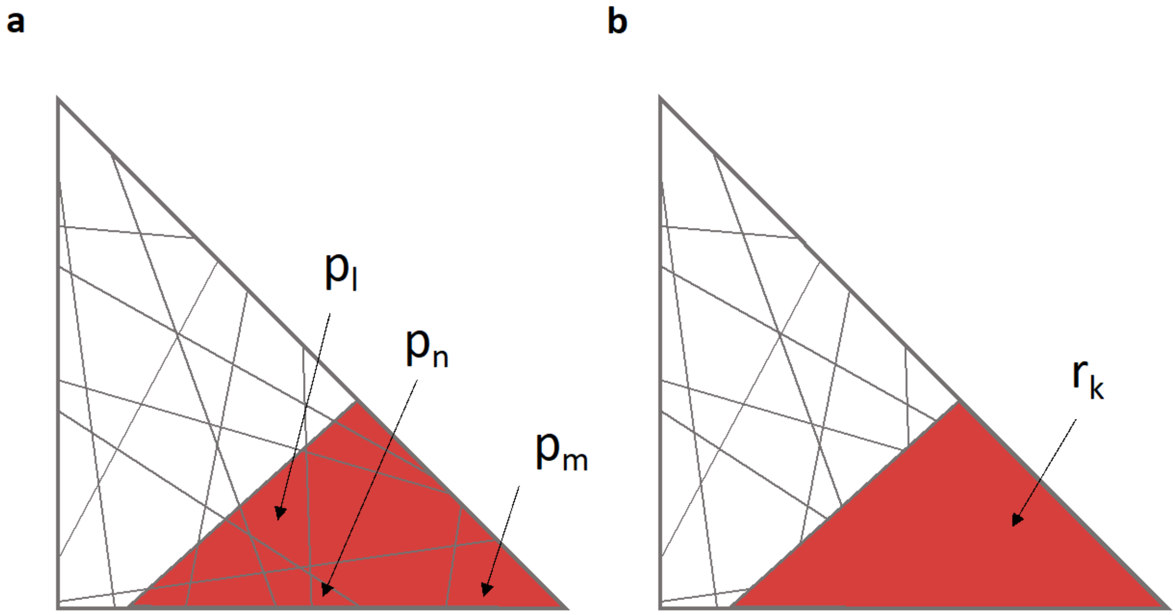

Supplementary Figure S5. From polygons to regions. Unification of the polygons in which the same method has the lowest average cost (**a,** shown in red) results in a more simplified view of the regions assigned to each predictor (**b**).

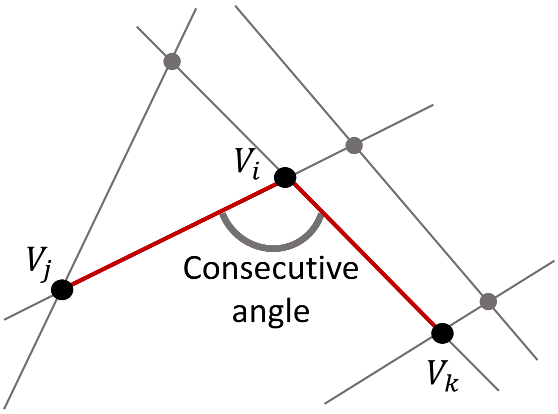

Supplementary Figure S6. Illustration of the consecutive angle formed by vertices v_i, v_j, and v_k.

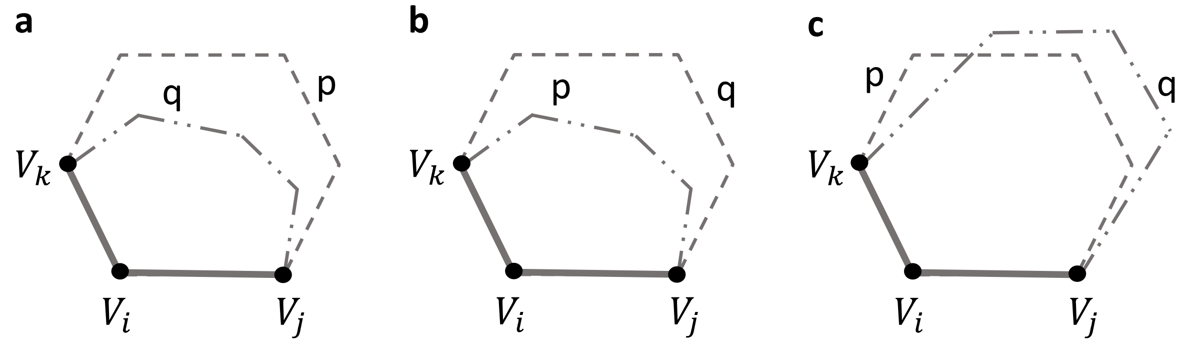

Supplementary Figure S7. Different situations for the relative location of polygons *p* and *q*. a, all the points in *q* are interior to *p*; **b,** all the points in *p* are interior to *q*; and **c,** both *p* and *q* have interior points from the other.

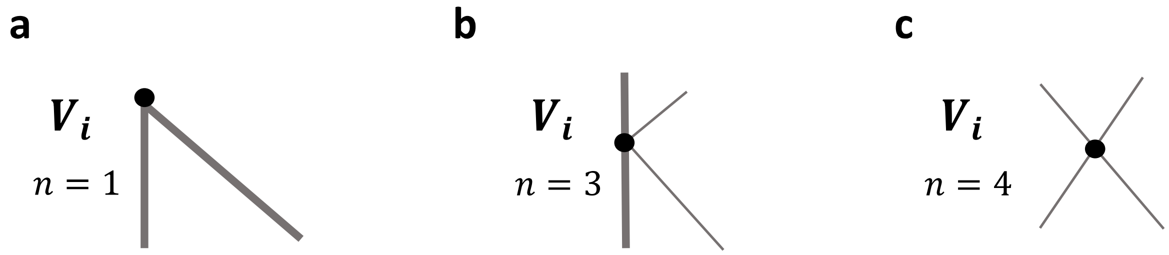

Supplementary Figure S8. Initial values of the counter of each vertex. In the three figures, thick grey lines correspond to the edges of the triangle ***T*** (the clinical space, see Appendix 1, Supplementary Materials), and thin grey lines correspond to the lines dividing ***T*** and associated with the different pair comparisons between methods. **a,** The vertex is one of the three triangle vertices. **b,** The vertex is the intersection between a triangle edge and the line associated with the comparison between two predictors**. c,** The vertex is the intersection between the lines associated to two different pair comparisons between predictors.

Supplementary Tables

Supplementary Table S1. The seventeen pathogenicity predictors used in this work. We provide the performance parameters required for the cost computations: sensitivity, specificity and coverage/reject rate. The column Output homogenization shows the correspondence between our pathogenic/benign states and the output of each predictor. We also list the decision cutoff when it was not provided by dbNSFP.

Predictor	Sensitivity	Specificity	Coverage/Reject rate	Output homogenization & decision cutoff
**CADD**	1	0.68	1/0	P >= 15<br>B < 15
**EVE**	0.92	0.85	0.43/0.57	P = Pathogenic<br>B = Benign
**LRT**	0.87	0.76	0.87/0.13	P = Deleterious<br>B = Neutral
**MetaLR**	0.87	0.88	0.99/0.01	P = Deleterious<br>B = Tolerated
**MetaSVM**	0.9	0.89	0.99/0.01	P = Deleterious<br>B = Tolerated
**MutPred**	0.95	0.87	1/0	P >= 0.5<br>B < 0.5
**MutationAssessor**	0.89	0.77	0.86/0.14	P = High, Medium<br>B = Low, Neutral
**MutationTaster**	0.98	0.74	0.99/0.01	P = Disease causing automatic, disease causing<br>B = Polymorphism, polymorphism automatic
**PMut**	0.84	0.87	0.85/0.15	P = Disease<br>B = Neutral
**PON-P2**	0.96	0.92	0.46/0.54	P = Pathogenic<br>B = Neutral
**PROVEAN**	0.9	0.84	0.97/0.13	P = Deleterious<br>B = Neutral
**Polyphen2_HDIV**	0.93	0.69	0.91/0.09	P = Probably damaging, possibly damaging<br>B = Benign
**Polyphen2_HVAR**	0.9	0.78	0.91/0.09	P = Probably damaging, possibly damaging<br>B = Benign
**REVEL**	0.92	0.94	1/0	P >= 0.5<br>B < 0.5

**SIFT**	0.93	0.75	0.97/0.03	P = Damaging<br>B = Tolerated
**SNAP2**	0.85	0.65	0.82/0.18	P = Effect<br>B = Neutral
**VEST4**	0.89	0.9	1/0	P >= 0.5<br>B < 0.5

Supplementary Table S2. Predictive performance of the thirteen pathogenicity predictors studied in Pejaver et al. [12]**.** In the first three columns we provide the performance parameters required for the cost computations (sensitivity, specificity and coverage/reject rate). In the last column, we give the score thresholds that define the pathogenic (P) and benign (B) classes for each predictor, according to Pejaver et al.'s (see Table 2 in [13]). The 'not classified' class pertains to variants with a prediction score falling between that of the pathogenic and benign classes. Note that for the pathogenic and benign classes we have unified the four levels (Supporting, Moderate, Strong, and Very Strong) provided by the authors.

Predictor	Sensitivity	Specificity	Coverage/Reject rate	Output homogenization & decision cutoff
**BayesDel**	0.916	0.906	0.797/0.203	P >= 0.130<br>B <= -0.180
**CADD**	0.912	0.831	0.782/0.218	P >= 25.3<br>B <= 22.7
**EA**	0.893	0.882	0.556/0.444	P >= 0.685<br>B <= 0.262
**FATHMM**	0.981	0.526	0.170/0.830	P <= -4.140<br>B >= 3.320
**GERP++**	0.000	1.000	0.237/0.763	B <= 2.700
**MPC**	1.000	0.000	0.192/0.808	P >= 1.360
**MutPred2**	0.902	0.921	0.773/0.227	P >= 0.737<br>B <= 0.391
**PhyloP**	0.890	0.792	0.618/0.382	P >= 7.367<br>B <= 1.879
**PolyPhen2**	0.895	0.833	0.550/0.450	P >= 0.978<br>B <= 0.113

**PrimateAI**	0.914	0.844	0.572/0.428	P >= 0.790<br>B <= 0.483
**REVEL**	0.923	0.913	0.732/0.268	P >= 0.644<br>B <= 0.290
**SIFT**	0.903	0.824	0.559/0.441	P <= 0.001<br>B >= 0.080
**VEST4**	0.928	0.902	0.723/0.277	P >= 0.764<br>B <= 0.449

Supplementary Table S3. Predictive performance of the rules for computational evidence in the two *ATM*-adapted versions of the ACMG/AMP guidelines. In the first three columns we provide the performance parameters required for the cost computations (sensitivity, specificity and coverage/reject rate). In the last column, we give the score thresholds that define the pathogenic (P) and benign (B) classes for each predictor, according to Clingen's expert panel [14] and according to Feliubadalo et al. [15]. The 'not classified' class pertains to variants with a prediction score falling between that of the pathogenic and benign classes.

Predictor	Sensitivity	Specificity	Coverage/Reject rate	Output homogenization & decision cutoff
**ClinGen**	1.000	0.985	0.825/0.175	P >= 0.733<br>B <= 0.249
**Feliubadaló**	0.922	0.945	0.948/0.052	P = Pathogenic<br>B = Benign

References

1. Vihinen, M. Problems in Variation Interpretation Guidelines and in Their Implementation in Computational Tools. *Mol. Genet. Genomic Med.* **2020**, *8*, e1206.
2. Hanczar, B. Performance Visualization Spaces for Classification with Rejection Option. *Pattern Recognit.* **2019**, *96*, 106984.
3. Chow, C.K. On Optimum Recognition Error and Reject Tradeoff. *IEEE Trans. Inf. Theory* **1970**, *6*, 41–46, doi:10.1109/TIT.1970.1054406.
4. Herbei, R.; Wegkamp, M.H. Classification with Reject Option. *Can. J. Stat.* **2006**, *34*, 709–721, doi:10.1002/cjs.5550340410.
5. Pepe, M.S. *The Statistical Evaluation of Medical Tests for Classification and Prediction*; Oxford University Press: Oxford, 2003;
6. Adams, N.M.; Hand, D.J. Comparing Classifiers When the Misallocation Costs Are

Uncertain. *Pattern Recognit.* **1999**, *32*, 1139–1147, doi:10.1016/S0031-3203(98)00154-X.

7. Hernández-Orallo, J.; Flach, P.; Ferri, C. A Unified View of Performance Metrics: Translating Threshold Choice into Expected Classification Loss. *J. Mach. Learn. Res.* **2012**, *13*, 2813–2869.
8. Lee, J.M. *Axiomatic Geometry*; American Mathematical Society: Providence, 2012;
9. Yaglom, I.M.; Boltyanskii, V.G. *Convex Figures*; Holt, Rinehart and Winston: New York, 1961;
10. de Berg, M.; Cheong, O.; van Kreveld, M.; Overmars, M. *Computational Geometry: Algorithms and Applications*; Springer, Ed.; 3rd Editio.; New York, 2008;
11. Fortuno, C.; James, P.A.; Young, E.L.; Feng, B.; Olivier, M.; Pesaran, T.; Tavtigian, S. V.; Spurdle, A.B. Improved, ACMG-Compliant, in Silico Prediction of Pathogenicity for Missense Substitutions Encoded by TP53 Variants. *Hum. Mutat.* **2018**, *39*, 1061–1069, doi:10.1002/humu.23553.
12. Pejaver, V.; Byrne, A.B.; Feng, B.; Radivojac, P.; Brenner, S.E.; Pejaver, V.; Byrne, A.B.; Feng, B.; Pagel, K.A.; Mooney, S.D.; et al. Calibration of Computational Tools for Missense Variant Pathogenicity Classification and ClinGen Recommendations for PP3 / BP4 Criteria. *Am. J. Hum. Genet.* **2022**, *109*, 2163–2177, doi:10.1016/j.ajhg.2022.10.013.
13. Pejaver, V.; Byrne, A.B.; Feng, B.-J.; Pagel, K.A.; Mooney, S.D.; Karchin, R.; O'Donnell-Luria, A.; Harrison, S.M.; Tavtigian, S. V.; Greenblatt, M.S.; et al. Evidence-Based Calibration of Computational Tools for Missense Variant Pathogenicity Classification and ClinGen Recommendations for Clinical Use of PP3/BP4 Criteria. *Am. J. Hum. Genet.* **2022**, *109*, 2163–2177.
14. HBOPC VCEP *ClinGen Hereditary Breast, Ovarian and Pancreatic Cancer Expert*

Panel Specifications to the ACMG/AMP Variant Interpretation Guidelines for ATM Version 1.1; 2022;

15. Feliubadaló, L.; Moles-Fernández, A.; Santamariña-Pena, M.; Sánchez, A.T.; López-Novo, A.; Porras, L.-M.; Blanco, A.; Capellá, G.; de la Hoya, M.; Molina, I.J.; et al. A Collaborative Effort to Define Classification Criteria for ATM Variants in Hereditary Cancer Patients. *Clin. Chem.* **2021**, *67*, 518–533, doi:10.1093/clinchem/hvaa250.
